# Insights into Phenolamides in Whole Grain Barley: Chemical Profile and Their Levels in Barley-Based Products

**DOI:** 10.1021/acs.jafc.5c04844

**Published:** 2025-08-19

**Authors:** Salar Hafez-Ghoran, Weixin Wang, Shengmin Sang

**Affiliations:** Laboratory for Functional Foods and Human Health, Center for Excellence in Post-Harvest Technologies, 3616 North Carolina Agricultural and Technical State University, North Carolina Research Campus, 500 Laureate Way, Kannapolis, North Carolina 28081, United States

**Keywords:** barley (*Hordeum vulgare* L.), phenolamides, hordatines, spermidine conjugates, molecular networking, targeted quantitation

## Abstract

Barley (*Hordeum vulgare* L.) is a nutrient-rich whole grain (WG) with pharmacological potential, partly attributed to its phenolamide content. Using ultrahigh-pressure liquid chromatography coupled with high-resolution electrospray mass spectrometry (UHPLC-HRESI-MS) integrated with Global Natural Products Social (GNPS) molecular networking, we identified 50 phenolamides in WG barley, including 13 agmatines, 14 spermidines, two putrescine conjugates, and 21 hordatines. Notably, we report for the first time two *p*-coumaroyl hydroxyagmatine derivatives (including a novel *cis*-isomer), three glycosylated spermidine conjugates, and two putrescine conjugates. Diagnostic MS/MS fragmentation enabled differentiation of 12 spermidine isomers, including CouCaf-Spd [Caf-(Put)-(*N* ^10^3AP)-Cou vs. Cou-(Put)-(*N* ^10^3AP)-Caf] and CafFer-Spd [Caf-(Put)-(*N* ^10^3AP)-Fer vs. Fer-(Put)-(*N* ^10^3AP)-Caf]. Targeted quantification of 44 phenolamides across various barley-based products (e.g., alcoholic and nonalcoholic beers, bread, flake, flour, hulled grains, and pearl grains) revealed product-specific distribution patterns, with hordatines and agmatine conjugates enriched in beers and spermidine conjugates more abundant in hulled grains and flours. Our findings expand the known diversity of barley phenolamides and offer a foundation for further investigation into their functional significance.

## Graphical abstract

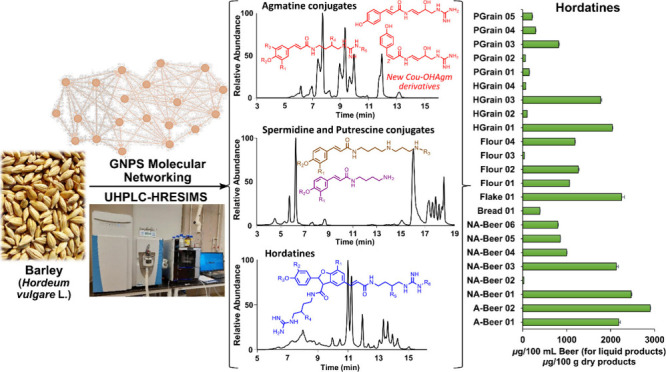

## 1 Introduction

Whole grain (WG) cereals are essential components of the human diet and are associated with reduced risks of chronic diseases including diabetes, obesity, cancer, and cardiovascular diseases. Among these, barley (*Hordeum vulgare* L.; Poaceae) stands out due to its long history of cultivation and its growing recognition over the past three to four decades as a health-promoting cereal.^1^ In addition to dietary fibers, vitamins, minerals, and essential amino acids, barely is rich in diverse bioactive phytochemicals. These include phenolamides (e.g., hydroxycinnamic acid conjugates of agmatine, spermidine, and putrescine), as well as hordatines, which are dimers of hydroxycinnamoyl agmatines, alongside flavonoids, procyanidins/proanthocyanidins, phytosterols, tocopherols, and folates, all contributing to its high antioxidant potential.^2^

Phenolamides are specialized metabolites that accumulate during plant development and in response to environmental stress. Besides reinforcing cell-wall integrity, they also play protective roles by deterring herbivores and inhibiting opportunistic pathogens. Although their pharmacological potential remains underexplored, phenolamides represent a compelling example of metabolic cross-talk between nitrogen and phenolic pathways.^3^ To date, only a limited number of barley-derived phenolamides have been purified^4,5^ or tentatively characterized without full structural elucidation.^6,7^ Beyond their antioxidant, anti-inflammatory, and antidiabetic properties,^8,9^ hordatines have also shown potential as anti-SARS-CoV-2 agents.^10^ Their precursors, *p*-coumaroyl agmatine and feruloylagmatine, have the capacity to detoxify methylglyoxal, a reactive carbonyl compound linked to diabetic complications, via mechanisms resembling those of metformin.^11^ Similarly, spermidine-conjugated hydroxycinnamic acids have exhibited various pharmacological effects, including antibiotic, anti-inflammatory, antitumor, anti-Alzheimer, antiviral, and antityrosinase properties.^12^ Nevertheless, the biological activities of many phenolamides remain insufficiently characterized.

There are few studies that reported hordatines in germinated barley,^4,6^ as well as their precursors and spermidines conjugates in malted barley and beer^7^ and hordatines and their precursors in beer brewing byproducts.^13^ However, the chemical profiles of these compounds in WG barley remain largely unexplored, which also leads to a lack of knowledge in terms of the content of these bioactive phenolamides in barley-based products, such as bread, flake, flour, hulled barley, pearl barley, and alcoholic and nonalcoholic beers. Only limited studies have addressed this topic, most notably, one reporting the average total hordatine content in 208 beers based on *p*-coumaric acid equivalents^14^ and another quantifying a single compound, hordatine A glucoside, in 10 barley cultivars (including six hulled barley and four hull-less barley).^5^

The Global Natural Products Social (GNPS) molecular networking is a mass spectrometry (MS)-based platform that can help researchers gain a comprehensive understanding of chemical and metabolic profiling through worldwide data sharing.^15^ GNPS employs advanced algorithms to analyze fragmentation patterns that might not be obvious using traditional methods and, therefore, to build molecular networks of compounds that share similar fragmentation patterns. With such as a powerful feature, it has recently been used to facilitate the identification of new bioactive compounds,^15^ such as steroidal saponins from oat bran.^16^

This study aims to identify new phytochemicals in WG barley and build up the chemical fingerprint using fragmentation patterns of phenolamides in both positive and negative modes in combination with GNPS. Furthermore, the quantification of phenolamides in various barley-based products is conducted using ultrahigh-pressure liquid chromatography coupled with high-resolution electrospray mass spectrometry (UHPLC-HRESI-MS).

## 2 Materials and Methods

### 2.1 Barley-Based Products, Chemicals, and Reagents

The alcoholic beers (*n* = 2 types) were obtained from a local supermarket (Food Lion, Kannapolis, NC), while the nonalcoholic beers (*n* = 6 types), bread (*n* = 1 type), flake (*n* = 1 type), flour (*n* = 4 types), hulled grain (*n* = 4 types), and pearl grain (*n* = 5 types) were ordered from Amazon. The ACS-grade, HPLC-grade, and LC-MS-grade solvents, such as methanol (MeOH), acetonitrile (MeCN), deionized water, and formic acid (FA), were obtained from Thermo Fisher Scientific (Petersburg, PA). Hordatines A–C (HA–HC), glycosylated hordatines A and B (HAG and HBG), and hordatine precursors, including *p*-coumaroyl agmatine (Cou-Agm) and feruloyl agmatine (Fer-Agm), and *p*-coumaroyl putrescine (Cou-Put) were either synthesized in-house or purified from barley extract.^4^

### 2.2 Preparation of Barley Samples

The dry forms of barley-based products, such as flakes, breads, flour, hulled grains, and pearl grains, were powdered using a SHARDOR Adjustable Coffee Grinder (Changsha Shardor Electrical Appliance Technology Co., Ltd., Changsha, China) at 20,000 × *g* for 30 s per sample. A total of 100 mg of each sample was extracted with 1 mL of 80% MeOH containing 0.1% FA at 20 °C using a homogenizer for 10 min. The samples were then sonicated for 10 min and centrifuged for 15 min at 15,000 × *g*. This extraction process was conducted twice. For the liquid forms of barley-based products, alcoholic and nonalcoholic beers, 1 mL of MeCN was added to 1 mL of each sample, followed by centrifugation for 15 min (15,000 × *g*) to remove any possible precipitate of proteins and undissolved material. Finally, all centrifuged and filtered samples were diluted 10-fold before UPLC-HRMS analysis.

### 2.3 Preparation of Standards

To quantify the phenolamides in various barley-based products, we prepared a stock solution of mixed barley standards. This included hordatines A–C (HA–HC), glycosylated hordatines A and B (HAG and HBG), and hordatine precursors, including *p*-coumaroyl agmatine (Cou-Agm) and feruloyl agmatine (Fer-Agm), and *p*-coumaroyl putrescine (Cou-Put).^4^ Preliminary analyses of barley samples indicated the highest concentrations of 5 μM for HA–HC, Cou-Agm, Fer-Agm, and Cou-Put and 50 μM for HAG and HBG, which were present in higher quantities. The main stock solution of mixed standards was then serially diluted 12-fold to have distinct concentration ranges for calibration curves: 2.44–5000 nM for the first set of standards (HA–HC, Cou-Agm, Fer-Agm, and Cou-Put) and 24.41–50000 nM for the second set (HAG and HBG).

To ensure reliable and accurate data, a quality control (QC) sample containing 1.56 μM each of HA–HC, Cou-Agm, Fer-Agm, and Cou-Put and 0.156 μM each of HAG and HBG was included every 12 samples to enhance the rigor of the quantitative analysis. The relative standard deviation (RSD%) for individual standards in the QC sample was measured as follows: HA (12.04%), HAG (11.30%), HB (11.86%), HBG (9.27%), HC (16.84%), Cou-Agm (11.30%), Fer-Agm (10.84%), and Cou-Put (7.76%). The concentration ranges (nM), linear equations (*y*), and their regressions (*R* ^2^) are presented in Table 1. The standard curves are shown in Supplementary Figure S1.

**Table 1:** Concentration Ranges (nM), Linear Equations (*y*), and Regression (*R* ^2^) for Barley Standards, Including HA–HC, HAG, HBG, Cou-Agm, Fer-Agm, and Cou-Put, Used for the Quantification Analysis of Phenolamides in Various Barley-Based Products^a^

Standard	Concentration range (nM)	*y* = *ax* + *b*	*R* ^2^
**Hordatine A**	2.44–156.25	*y* = 883982*x* + 575997	0.9993
	156.25–5000	*y* = 529964*x* + 1 × 10^8^	0.9950
**Hordatine B**	2.44–39.06	*y* = 824491*x* – 239429	0.9999
	39.06–5000	*y* = 520012*x* + 8 × 10^7^	0.9946
**Hordatine C**	2.44–78.13	*y* = 576729*x* – 284424	0.9999
	78.13–5000	*y* = 407546*x* + 5 × 10^7^	0.9969
**Hordatine A Glu**	24.41–781.25	*y* = 408619*x* + 1 × 10^7^	0.9962
	390.63–25000	*y* = 250675*x* + 2 × 10^8^	0.9985
**Hordatine B Glu**	24.41–781.25	*y* = 321537*x* + 7 × 10^6^	0.9985
	390.63–25000	*y* = 206181*x* + 2 × 10^8^	0.9975
**Cou-Agm**	2.44–19.53	*y* = 773517*x* + 33346	1.0000
	19.53–312.50	*y* = 716425*x* + 3 × 10^6^	0.9993
	312.50–5000	*y* = 344795*x* + 2 × 10^8^	0.9829
**Fer-Agm**	2.44–78.13	*y* = 480796*x* – 190583	0.9999
	39.06–2500	*y* = 358668*x* + 3 × 10^7^	0.9949
**Cou-Put**	2.44–78.13	*y* = 414013*x* – 650839	0.9998

a Commercially available barley-based products: Alcoholic beers (*n* = 2 types), nonalcoholic beers (*n* = 6 types), bread (*n* = 1 type), flake (*n* = 1 type), flour (*n* = 4 types), hulled grain (*n* = 4 types), and pearl grain (*n* = 5 types).

### 2.4 UHPLC-HR-ESI-MS Analysis

An ultrahigh pressure liquid chromatograph (UHPLC) coupled with an Orbitrap Q Executive Plus (QE+) accurate mass spectrometer (Thermo Fisher Scientific) was used to quantify phenolamides in barley-based products (*n* = 20). The UHPLC was equipped with an autosampler (VANQUISH model), a column compartment (VANQUISH model), binary pumps (VANQUISH model), and a degasser (VANQUISH model). Chromatographic separation was obtained using a reverse-phase Hypersil GOLD analytical column (100 × 1.0 × 1.9 μm^3^) at a temperature of 40 °C, with a solvent flow rate of 0.2 mL/min and injection volume of 3 μL. The gradient elution consisted of eluent A (water + 0.1% FA) and eluent B (MeCN + 0.1% FA). The gradient elution profile was as follows: 2% B (0–1.0 min), 2–15% B (1.0–1.5 min), 15% B (1.5–6.0 min), 15–30% B (6.0–13.0 min), 30–50% B (13.0–16.0 min), 50–100% B (16.0–17.0 min), 100% B (17.0–21.0 min), and 2% B (21.0–21.5 min).

Mass analyses were performed using a QE+ accurate mass spectrometer equipped with an electrospray ionization (ESI) source, operating in negative and positive ionization modes. Collision energies (CE) were set at 20, 35, and 50 eV to optimize fragmentation and maximize transmission of the desired product ions. The resolutions were 70,000 (full mass) and 17,500 (ddMS^2^), with scan range of 100 to 1200 *m*/*z*. The MS operating conditions included sheath gas (N_2_) flow at 50 L/min, auxiliary gas (N_2_) flow at 5 L/min, sweep gas (N_2_) flow rate at 3 L/min; capillary temperature at 350 °C; auxiliary gas heater temperature at 365 °C; spray current at 12.5 μA; and spray voltage at 1.0 kV. LC-HRESI-MS data were acquired and processed by using Xcalibur software (Thermo Fisher Scientific).

### 2.5 Global Natural Products Social Molecular Networking (GNPS) Analysis

The UHPLC-HRESI-MS raw data of WG barley extract, obtained in positive and negative modes, were converted to the mzML format using the MSconvert package (Version 3.0.21271, Proteowizard Software Foundation, USA). This format ensures compatibility with GNPS, and the data were uploaded to the GNPS platform using WinSCP Version 6.3.6. The data were then subjected to GNPS analysis (Molecular networking) through the online platform at https://gnps.ucsd.edu/ProteoSAFe/static/gnps-splash.jsp. The resultant networks were analyzed online.^15^

### 2.6 Data Analysis

The quantification of phenolamides in barley products (*n* = 23 types) was performed in two independent samples. The results were expressed as mean ± SD.

## 3 Results and Discussion

### 3.1 Identification of Two *p*-Coumaroyl Hydroxyagmatine Derivatives

In order to establish the chemical profile of WG barley, a GNPS-guided strategy was applied. As a starting point for molecular networking, *p*-coumaroyl agmatine (Cou-Agm; **1**) (Figure 1A), which serves as the precursor for hordatines A and B, was selected. Using a stepped normalized collision energy (NCE: 20, 35, 50%), molecular networking delineated a clear relationship between Cou-Agm (**1**) and an analogue (**2**) with a 4 Da lower mass. Both compounds featured the diagnostic *p*-coumaroyl fragment (*m*/*z* 147.0441; C_9_H_7_O_2_). In the case of compound **2**, higher-resolution MS^1^ at NCE 20% revealed a molecular formula of C_14_H_19_O_3_N_4_ (*m*/*z* 291.1448), while the MS^2^ spectrum exhibited a prominent ion at *m*/*z* 273.1342 [M – H_2_O + H]^+^, corresponding to dehydration during ionization (C_14_H_17_O_2_N_4_) (Figure 1C). The LC-HRMS chromatogram displayed two peaks at 7.46 and 9.81 min (Figure 1B), both exhibiting the same molecular formula and mass, indicative of isomeric forms. In the positive ion mode, the fragmentation patterns of compounds **1** and **2** showed different behaviors, although they were similar in the *p*-coumaroyl part. Compound **1** lost 17 Da ([M – NH_3_ + H]^+^),^17^ while compound **2** underwent two successive neutral losses of 18 Da; yielding *m*/*z* 255.1236; C_14_H_15_ON_4_), followed by a neutral loss of 42 Da (CH_2_N_2_), which is characteristic of the terminal part of the agmatine chain, yielding *m*/*z* 213.1022 (C_13_H_13_ON_2_). Moreover, compound **2** exhibited a loss of 126 Da (C_5_H_10_N_4_), which is 4 Da less than that of the agmatine fragment (130 Da). This observation suggested that the agmatine moiety in compound **2** may undergo two oxidative modifications, following the initial loss of a water molecule. This formed two additional double bonds within the putrescine chain (Figure 1D). To further confirm this hypothesis, the fragmentation pattern of compound **2** was also analyzed in the negative ion mode. The precursor ion appeared at *m*/*z* 271.1192 [M – H_2_O – H]^−^, consistent with the loss of a water molecule. In this mode, compound **2** displayed two major fragments: a neutral loss of 120 Da, corresponding to a 4-vinylphenol residue (yielding *m*/*z* 156.0621, C_6_H_7_ON_4_), and a neutral loss of 152 Da, indicating the presence of a carbonyl group and an oxidized agmatine residue (yielding *m*/*z* 119.0499, C_8_H_7_O from the *p*-coumaroyl part) (Figure 1E). Collectively, these findings identified compound **2** as *p*-coumaroyl hydroxydehydroagmatine (Cou-OHdhAgm), newly reported in WG barley.

**Figure 1:**
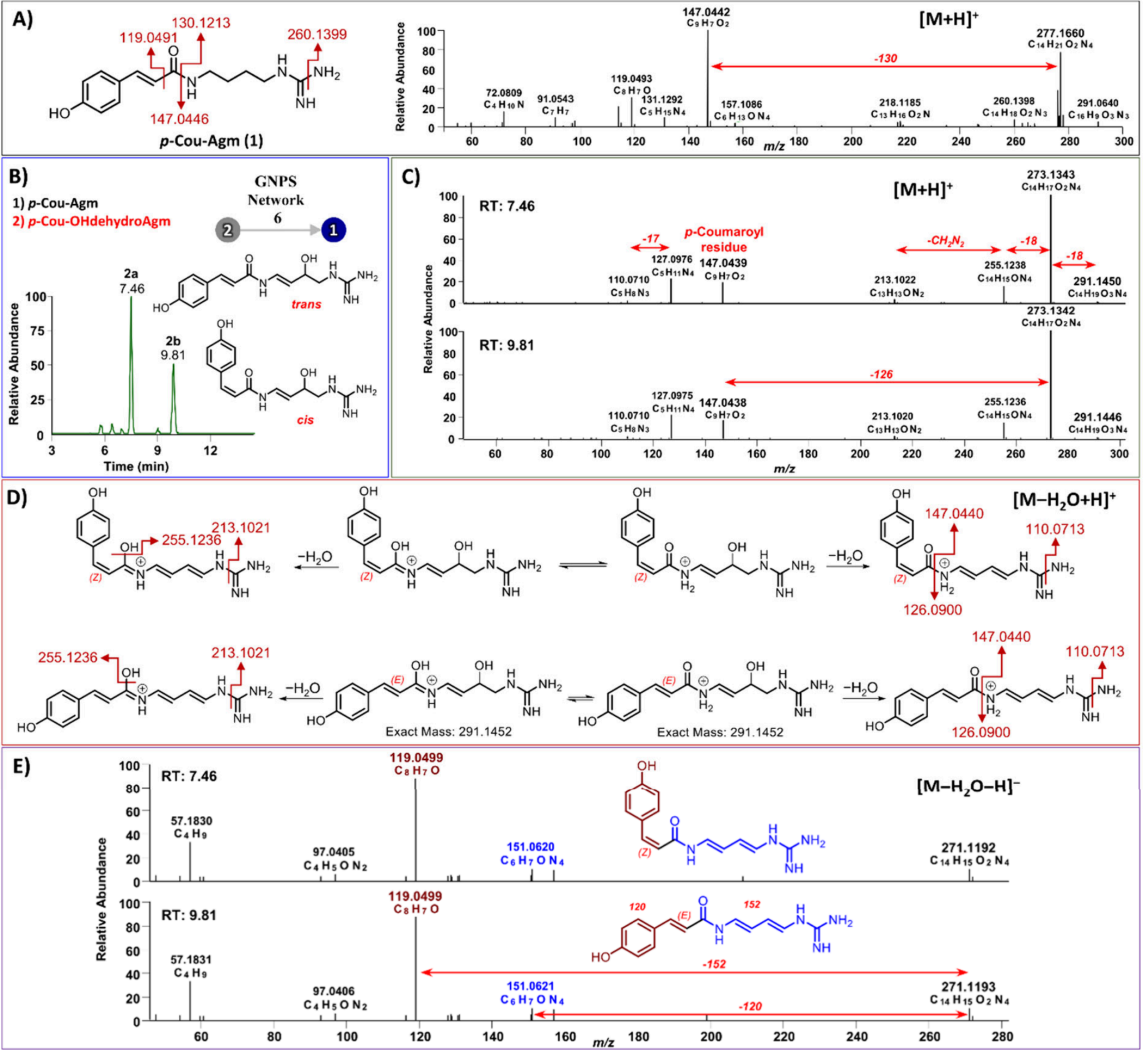
GNPS-guided identification of *p*-coumaroyl hydroxydehydroagmatine (Cou-OHdhAgm; **2**). (**A**) Chemical structure and MS^2^ spectrum of *p*-coumaroyl agmatine (**1**) acquired in positive ion mode under stepped normalized collision energies (NCE: 20, 35, 50%). (**B**) Structures and extracted ion chromatograms of Cou-OHdhAgm isomers: *cis* isomer (**2a**, at 7.46 min) and *trans* isomer (**2b**, at 9.81 min). (**C**) MS^2^ spectrum of Cou-OHdhAgm isomers in positive ion mode at NCE 20%, (**D**) Proposed fragmentation pattern of Cou-OHdhAgm isomers under positive ionization, (**E**) MS^2^ spectrum of Cou-OHdhAgm isomers in negative ion mode under stepped NCE (20, 35, 50%).

According to the literature, plants are more likely to contain both the *cis* and the *trans* isomers of *p*-coumaric acid. For example, a phytochemical investigation of *Grewia optiva* resulted in the isolation of *cis*-tiliroside and *trans*-tiliroside, bearing a *p*-coumaroyl moiety (*p*-Cou) in their structures.^18^ Sigurdson et al. evaluated the effects of the *cis*/*trans* configuration of the acylating group (*p*-Cou) on the HPLC profile of anthocyanins, particularly delphinidin-3-rutinoside-5-glucoside (De-3-Rut-5-Glu) and petunidin-3-rutinoside-5-glucoside (Pt-3-Rut-5-Glu), which were isolated from the skin of eggplants and black goji fruits. They observed that the *trans* isomer of both compounds (De-3-*trans*-*p*-Cou-Rut-5-Glu and Pt-3-*trans*-*p*-Cou-Rut-5-Glu) appear at later retention times than their corresponding *cis* isomers (De-3-*cis*-*p*-Cou-Rut-5-Glu and Pt-3-*cis*-*p*-Cou-Rut-5-Glu), when using a C18 column.^19^ Similarly, the *cis*/*trans* isomers of delphinidin 3-[4-(*p*-coumaroyl)-l-rhamnosyl­(1→6)­glucoside]-5-glucoside were isolated from eggplant, and a reverse-phase HPLC chromatogram revealed that the *cis* form eluted earlier than the *trans* from.^20^ Taking these observations into account, the presence of two peaks at different retention times (7.46 and 9.81 min) further supported that Cou-OHdhAgm may potentially exist as two isomers, with *cis*-Cou-OHdhAgm (**2a**) at 7.46 min, which is a new compound, and *trans*-Cou-OHdhAgm (**2b**) at 9.81 min, which has previously been described as a phytoalexin in germinated wheat leaves infected with *Bipolaris sorokiniana* ^21^ (Figure 1B). Considering that no NMR data of these two isoforms have been reported, it is worthwhile to get authentic standards of these two isomers and further confirm their structures using NMR.

### 3.2 Chemical Profile of Agmatine-Containing Phenolamides in Whole Grain Barley

Agmatine-containing phenolamides are predominant in barley^7^ and are also present in lower quantities in rye^22^ and wheat^23^ grains. However, their detailed chemical profile has yet to be explored. In addition to the connection between *p*-coumaroyl agmatine (**1**) and *p*-coumaroyl hydroxydehydroagmatine (Cou-OHdhAgm, **2**), further analysis of agmatine-containing phenolamides by GNPS in positive ion mode revealed limited connections between compounds. Consequently, the chemical profile of agmatine-containing phenolamides was examined using knowledge-based approaches, involving a search for known compounds reported in the literature with their structures confirmed by analyzing HR-ESI-MS/MS data. The chemical structures, including agmatines (**1**, **9**, **11**), hydroxyagmatines (**2a/2b**, **6**, **8**), methylated agmatines (**10**, **12**), and sugar-containing agmatines (**3**–**5**, **7**), along with their corresponding LC/MS profiles, are shown in Figure 2. It is worth mentioning that the levels of *p*-coumaroyl- and feruloyl hydroxyagmatine (**6** and **8**) were found to be higher than their respective non-hydroxylated counterparts (i.e., **1** and **11**).

**Figure 2:**
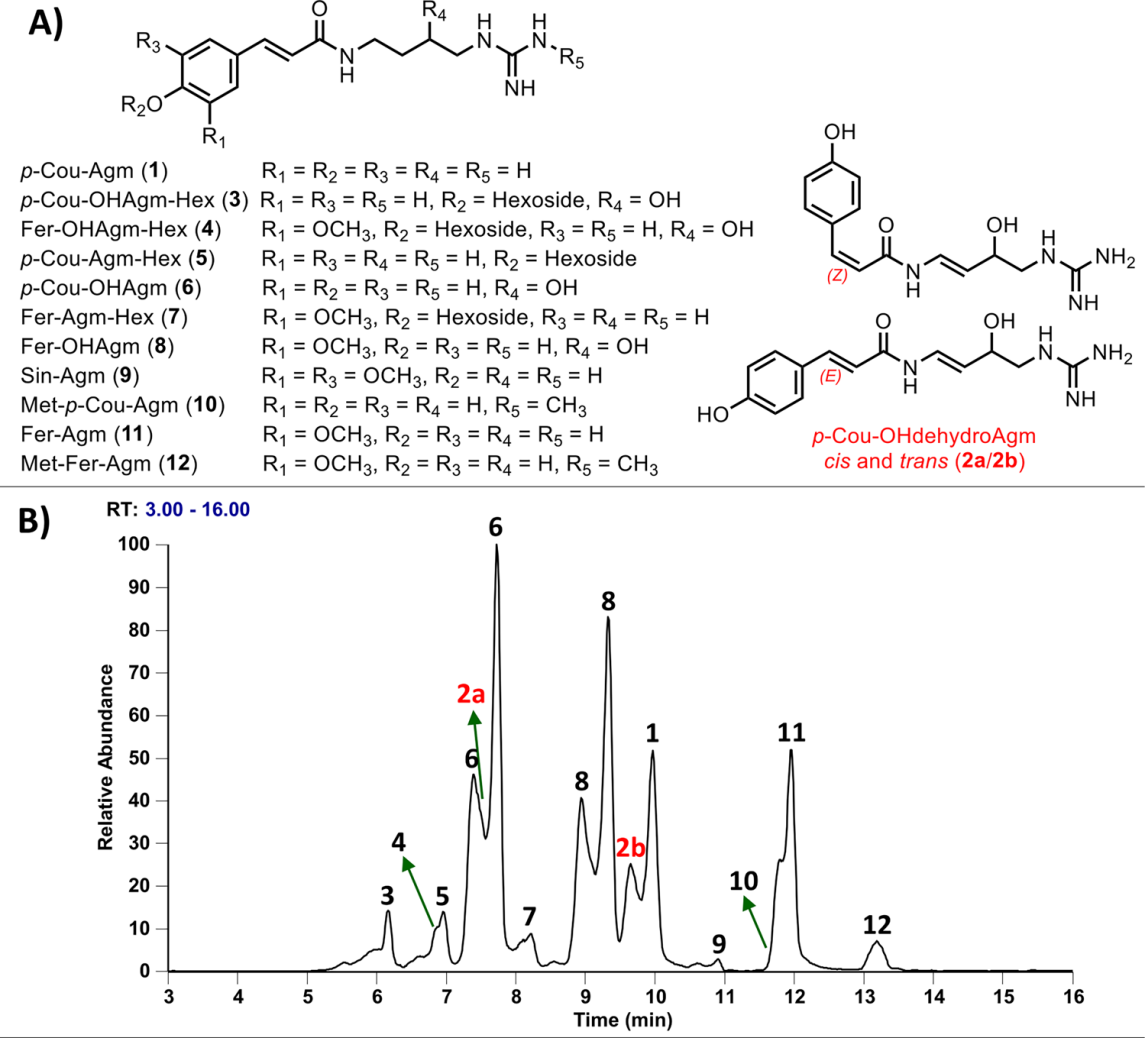
Chemical structures of major agmatine-containing phenolamides **1**–**12** identified in whole grain barley (**A**) and their chemical profile (**B**) in positive ion mode, based on selected ions.

### 3.3 GNPS Molecular Networking of Spermidine-Containing Phenolamides

To expand the network of barley phenolamides, Cou-Agm (**1**) was selected as the starting point for GNPS analysis in the negative ion mode. This analysis revealed a molecular network of spermidine conjugates (Figure 3A), suggesting structural interconnections through shared fragmentation pathways. The characteristic fragment ions corresponding to three hydroxycinnamic acid derivatives were identified as follows: *p*-coumaroyl residue at *m*/*z* 119.0497 (C_8_H_7_O), caffeoyl residue at *m*/*z* 135.0446 (C_8_H_7_O_2_), and feruloyl residue at *m*/*z* 149.0603 (C_9_H_9_O_2_) (Figure 3B). The network between *p*-Cou-Agm (**1**) and spermidine conjugates di-*p*-coumaroyl spermidine (diCou-Spd; **13**) and *p*-coumaroyl feruloyl spermidine (CouFer-Spd; **14**) was established based on their shared *p*-coumaroyl fragment ion (*m*/*z* 119.0497; C_8_H_7_O). This network extended from CouFer-Spd (**14**) to feruloyl caffeoyl spermidine (FerCaf-Spd; **15**), diferuloyl spermidine (diFer-Spd; **16**), feruloyl hydroxyagmatine (Fer-OHAgm; **8**), and feruloyl agmatine (Fer-Agm; **11**), all of which share the feruloyl fragment ion (*m*/*z* 149.0609; C_9_H_9_O_2_). Additionally, compound **15**, which contains both feruloyl and caffeoyl moieties, also shares the caffeoyl fragment ion (*m*/*z* 135.0446; C_8_H_7_O_2_) with dicaffeoyl spermidine (diCaf-Spd; **17**), suggesting a dual interaction between feruloyl and caffeoyl derivatives in these polyamine conjugates. The fragmentation pattern of compound **17** was found to be similar to the findings of Kang et al., who identified the same compound in red, brown, and white sorghum whole grains.^24^ Therefore, this compound is most likely a linear spermidine diconjugate with caffeoyl moieties attached at the *N* ^1^ and *N* ^10^ positions. Overall, the observed molecular network supports the hypothesis that *p*-coumaroyl, caffeoyl, and feruloyl residues play critical roles in the structural organization and fragmentation behavior of these spermidine-containing phenolamides.

**Figure 3:**
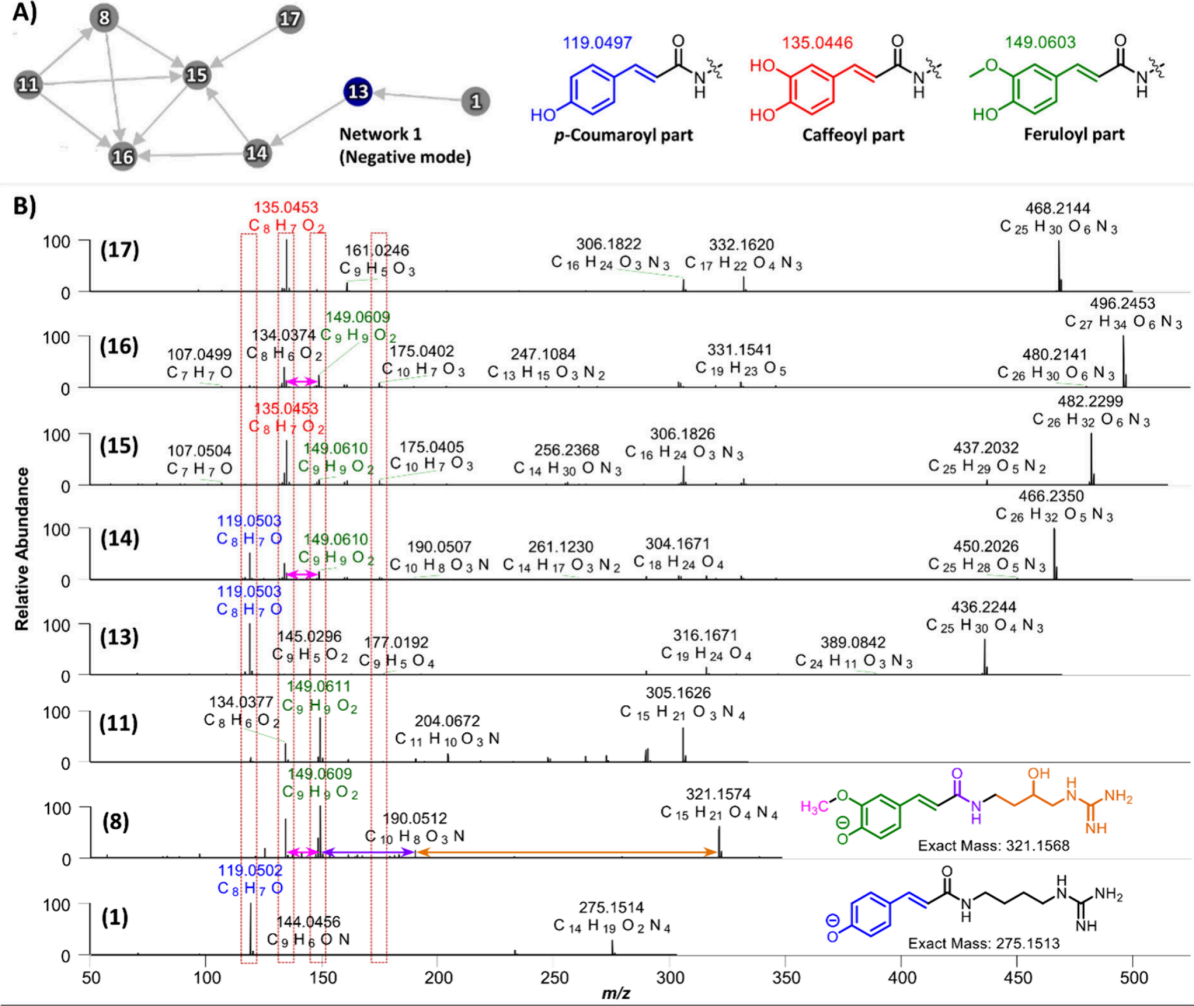
GNPS analysis of spermidine-containing phenolamides in whole grain barley in negative ion mode. (**A**) Molecular networks of *p*-Cou-Agm (**1**), Fer-OHAgm (**8**), Fer-Agm (**11**), diCou-Spd (**13**), CouFer-Spd (**14**), FerCaf-Spd (**15**), diFer-Spd (**16**), and diCaf-Spd (**17**); (**B**) MS^2^ fragmentation pattern of spermidine and agmatine conjugates in negative ion mode.

To further expand the profile of spermidine-containing phenolamides, GNPS molecular networking was also analyzed in positive ion mode, which showed simpler networks (Figure 4A). However, this analysis led to the identification of five additional spermidine-containing phenolamides, including caffeoyl spermidine (Caf-Spd; **18**), feruloyl spermidine hexoside (Fer-Spd Hex; **19**), feruloyl spermidine (Fer-Spd; **20**), dicaffeoyl spermidine hexoside (diCaf-Spd Hex; **21**), and *p*-coumaroyl caffeoyl spermidine (CouCaf-Spd; **22**). Notably, the hexoside-containing spermidines (**19** and **21**) are reported for the first time in WG barley.

**Figure 4:**
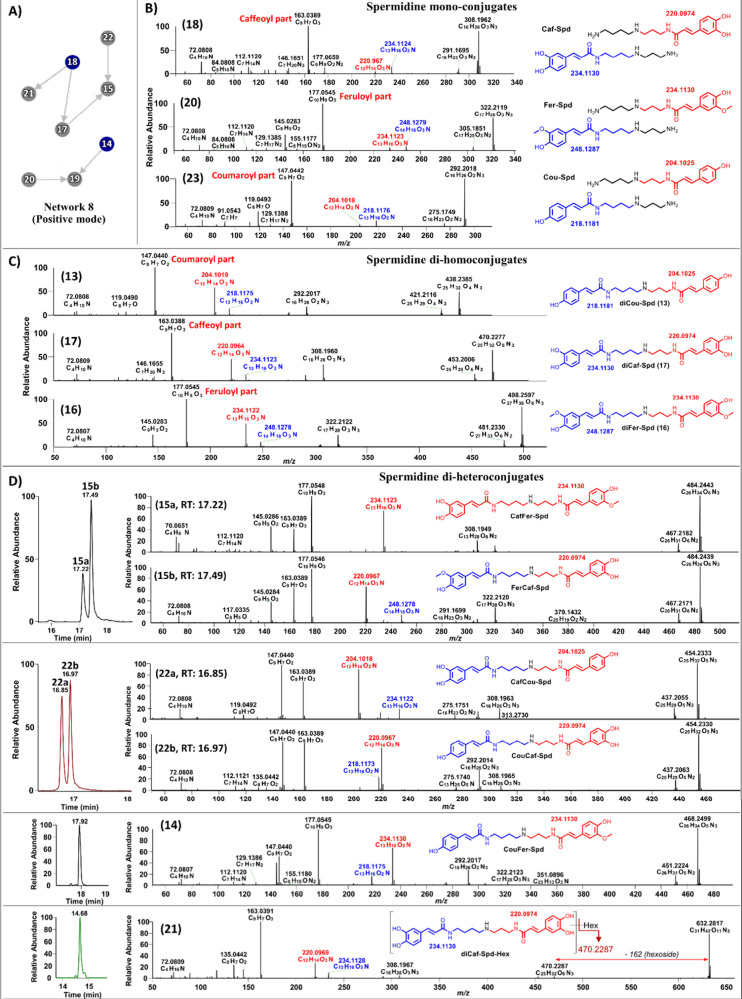
GNPS analysis of spermidine-containing phenolamides in whole grain barley in positive ion mode. (**A**) Molecular network of CouFer-Spd (**14**), FerCaf-Spd (**15**), diCaf-Spd (**17**), Caf-Spd (**18**), Fer-Spd Hex (**19**), Fer-Spd (**20**), diCaf-Spd Hex (**21**), and CouCaf-Spd (**22**). (**B**) Differentiation of spermidine monoconjugates (compounds **18**, **20**, and **23**) into two structural forms based on their fragmentation patterns. (**C**) Characteristic fragment ions observed in spermidine dihomoconjugates (compounds **13**, **16**, and **17**). (**D**) Distinct fragmentation peaks corresponding to spermidine diheteroconjugates (compounds **14**, **15a**/**15b**, **21**, and **22a**/**22b**).

### 3.4 Characteristic Fragment Ions for Structure Confirmation of Spermidine-Containing Phenolamides

GNPS molecular networking identified a variety of spermidine conjugates, including monoconjugates (compounds **18** and **20**), diconjugates (compounds **13**–**17** and **22**), and hexoside-containing conjugates (compounds **19** and **21**) (Figures 3A and 4A). Building upon these results, we further identified two structurally related compounds: *p*-coumaroyl spermidine (Cou-Spd; **23**) and feruloyl caffeoyl spermidine hexoside (FerCaf-Spd Hex; **24**). While monoconjugates (compounds **18**, **20**, and **23**), dihomoconjugates (compounds **13**, **16**, and **17**), and diheteroconjugates (compounds **14**, **15**, and **22**) have previously been reported in barley and beer,^7^ this is the first report to completely elucidate their structures based on detailed analysis of their fragment ions. Additionally, this is also the first report of hexoside-containing spermidine conjugates (compounds **19**, **21**, and **24**).

Due to the asymmetric nature of spermidine, which possesses two terminal NH_2_ groups, one associated with putrescine and the other with the *N* ^10^-(3-aminopropyl) (*N* ^10^3AP) portion, hydroxycinnamic acid derivatives can form amide bonds at either end. As a result, *p*-coumaroyl, caffeoyl, and feruloyl groups may attach to either the NH_2_-terminus of putrescine (*N* ^1^) or the NH_2_-terminus of *N* ^10^3AP (*N* ^10^). Although several spermidine-containing phenolamides have been previously reported in WG barley, the exact linkage sites of hydroxycinnamic acids on the spermidine backbone have not been fully elucidated.

By analyzing MS^2^ fragmentation patterns, we identified characteristic fragment ions that provide insight into the structural isomerism of these conjugates. In monoconjugated spermidines, including Caf-Spd (**18**), Fer-Spd (**20**), and Cou-Spd (**23**), fragmentation resulted in pairs of diagnostic ions at *m*/*z* 220 and 234 (for **18**), 234 and 248 (for **20**), and 204 and 218 (for **23**) (Figure 4B), suggesting the presence of two isomeric forms in barley. This pattern was also observed in dihomoconjugated spermidines, including diCou-Spd (**13**), diFer-Spd (**16**), and diCafSpd (**17**), which show similar dual-fragmentation signatures (Figure 4C). To explore this further, we examined the fragmentation patterns of spermidine diheteroconjugates, including CafFer-Spd (**15a**/**15b**) and CafCou-Spd (**22a**/**22b**) (Figure 4D). Although the GNPS network (Figure 4A) showed a single node for each compound, the LC-MS chromatograms exhibited two closely spaced peaks, indicating the presence of structural isomers. Detailed analysis of the fragmentation patterns enabled the structural elucidation of these isomers. For CafFer-Spd (**15**), the fragment ion at *m*/*z* 234.1123 (C_13_H_16_O_3_N) indicated attachment of the feruloyl group to the *N* ^10^3AP moiety, assigning compound **15a** (*t*_R_ = 17.22 min) the structure Caf-Put-(*N* ^10^3AP)-Fer. Conversely, in compound **15b** (*t*_R_ = 17.49 min), the fragment ion at *m*/*z* 220.0967 (C_12_H_14_O_2_N) suggested feruloylation at the putrescine moiety. This fragmentation pattern was consistent with the structure of Fer-(Put)-(*N* ^10^3AP)-Caf. A similar strategy was applied to CafCou-Spd (**22**). In compound **22a** (*t*_R_ = 16.85 min), the fragment ion at *m*/*z* 204.1018 (C_12_H_14_O_2_N) supported the presence of a *p*-coumaroyl group on the *N* ^10^3AP moiety, yielding the structure Caf-(Put)-(*N* ^10^3AP)-Cou. In contrast, compound **22b** (*t*_R_ = 16.97 min) produced a fragment ion at *m*/*z* 220.0967 (C_12_H_14_O_3_N), indicating caffeoyl substitution on the *N* ^10^3AP moiety. This fragmentation pattern was consistent with the structure of Cou-(Put)-(*N* ^10^3AP)-Caf (Figure 4D). Using the same analytical strategy, CouFer-Spd (**14**) was confirmed as Cou-Put-(*N* ^10^3AP)-Fer, based on the observation of a single chromatographic peak and the presence of characteristic fragment ions at *m*/*z* 234.1123 and 218.1175 (Figure 4D). Similarly, the structure of the sugar-containing spermidine dihomoconjugate (diCafSpd-Hex; **21**) was confirmed as shown in Figure 4D.

### 3.5 Chemical Profile of Spermidine-Containing Phenolamides

Using a combination of GNPS molecular networking, knowledge-based targeted searching, and characteristic fragment ions, we confirmed the structures of 12 spermidine-containing phenolamides (Figure 5A) and generated their chemical profiles (Figure 5B). As shown in Figure 5B, the chemical profile of spermidine conjugates revealed that monohydroxycinnamic acid derivatives, including Caf-Spd (**18**), Fer-Spd-Hex (**19**), Fer-Spd (**20**), and Cou-Spd (**23**), appear prominently at the beginning of the chromatogram, with compound **20** representing the highest peak. Toward the end of the chromatogram (*t*_R_ = 14.5–18.5 min), spermidine diconjugates and their hexoside-containing derivatives (compounds **13**–**17**, **21**, **22**, and **24**), were observed (Figure 5B).

**Figure 5:**
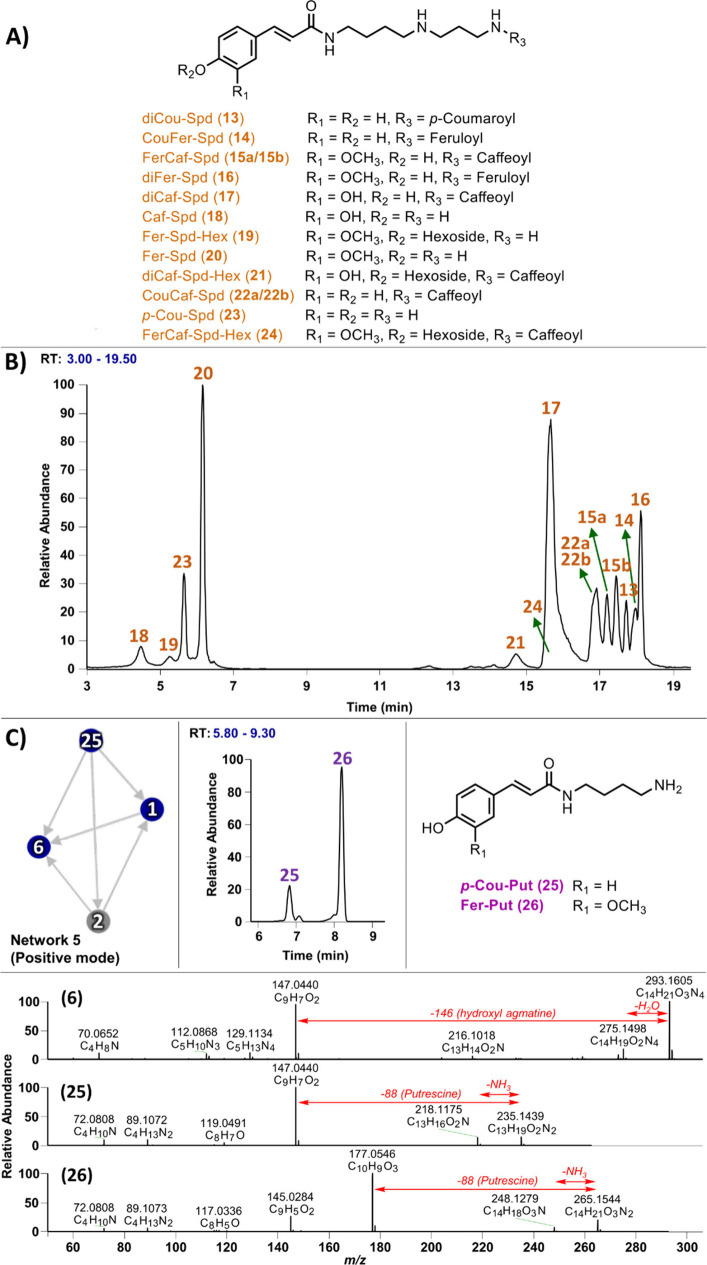
Chemical structures and chemical profiles of spermidine-containing and putrescine-containing phenolamides. (**A**) The chemical structures of spermidine-containing phenolamides **13**–**24** identified in whole grain barley. (**B**) The LC-HRMS chemical profile of spermidine-containing phenolamides in the positive ion mode, based on selected ions. (**C**) GNPS molecular networking, the LC-HRMS chemical profile (based on selected ions), chemical structures, and MS/MS spectra of putrescine-containing phenolamides **25** and **26** identified in whole grain barley (CouAgm **1**, Cou-OHdhAgm **2**, Cou-OHAgm **6**, Cou-Put **25**).

### 3.6 Chemical Profile of Putrescine-Containing Phenolamides

GNPS molecular networking facilitated the annotation of *p*-coumaroyl putrescine (Cou-Put; **25**) in WG barley. The molecular network of compound **25** clustered with CouAgm (**1**), *cis*/*trans*-Cou-OHdhAgm (**2**), and Cou-OHAgm (**6**) (Figure 5C), all of which share the *p*-coumaroyl moiety. In addition, conventional analysis confirmed the presence of feruloyl putrescine (Fer-Put; **26**). Compared to agmatine- and spermidine-based phenolamides, both Cou-Put (**25**) and Fer-Put (**26**) were detected in relatively lower quantities (Figure 5C). While Cou-Put (**25**) has been previously reported in germinated barley^4^ and Fer-Put (**26**) in beer,^7^ this study presents the first evidence of these two compounds in WG barley.

### 3.7 Ionization and Fragmentation Pattern of Hordatines

Hordatines, which are dimers of hydroxycinnamic acid agmatines, constitute a class of barley-specific phytochemicals. Hordatines A, B, and C and their corresponding glucosides are the major hordatines in barley. Pihlava proposed the term hordatines A_1_ and A_2_ for those hordatines that contain one and two hydroxyl groups in their agmatine residues, respectively, which also applies to hordatines B and C.^7^ However, the detailed chemical profile of hordatines is insufficiently characterized.

To address this knowledge gap, we applied the same GNPS molecular networking strategy as that used for spermidine conjugates to develop the chemical profile of hordatines. Prior to GNPS analysis, we first examined the ionization behavior and fragmentation patterns of the hordatines. Hordatine A_2_ (**27**), a homodimer of two units of *p*-coumaroyl hydroxyagmatine (Cou-OHAgm), was selected as an example to elucidate the fragmentation patterns of hordatines in both positive and negative ion modes. In positive ionization mode, this compound exhibited two ion types: singly charged [M + H]^+^ at *m*/*z* 583.2984 and doubly charged [M + 2H]^2+^ at *m*/*z* 292.1530 (Figure 6A). Although the singly charged ion was detected at a lower intensity (NL: 1.11 × 10^6^), it provided more informative fragmentation data. Successive neutral losses of 146 Da (hydroxyagmatine residue) led to the production of ions at *m*/*z* 437.1815 and *m*/*z* 291.0652. Additional fragmentation of the hydroxyagmatine moiety resulted in characteristic losses of 17 (−NH_3_), 25 (−CN+H), 18 (−H_2_O), and 86 Da (−C_4_H_10_N_2_). In contrast, the doubly charged ion displayed much higher intensity in both the LC chromatogram (NL: 1.77 × 10^7^) and MS^1^, making it advantageous for detecting hordatines. However, its fragmentation pattern was approximately half that of the singly charged ion (Figure 6A), suggesting a limited structural elucidation value.

**Figure 6:**
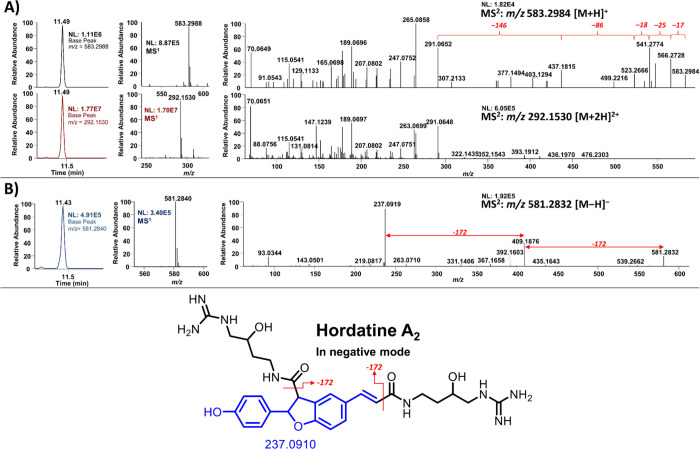
Ion chromatograms and MS^1^ and MS^2^ spectra of hordatine A_2_ (**27**) in both positive and negative ion modes. (**A**) Singly charged and doubly charged ions of hordatine A_2_ in positive ion mode, (**B**) Singly charged ion of hordatine A_2_ in negative ion mode.

In the negative ion mode, hordatine A_2_ (**27**) showed a deprotonated molecular ion at *m*/*z* 581.2840 (calculated: 581.2842), corresponding to the molecular formula C_28_H_37_N_8_O_6_^–^. A diagnostic peak at *m*/*z* 237.0919, corresponding to the oxidized *p*-coumaric acid dimer, indicates that this compound belongs to type A hordatines.^25^ Although the molecular ion intensity of MS^1^ was lower in the negative mode compared to both singly and doubly charged ions observed in the positive mode, the negative mode produced a few major structurally informative fragment ions with high intensity and fewer smaller fragment ions than those observed from the singly charged ion in the positive mode. Notably, two successive losses of 172 Da revealed the core backbone of hordatine A (Figure 6B). Therefore, while the positive mode, especially with doubly charged ions, offers greater sensitivity for detection, the negative ion mode is better suited for structural elucidation of hordatines.

### 3.8 GNPS Molecular Networking of Hordatines

Based on the fragmentation patterns of hordatines, GNPS molecular networking analysis of WG barley was performed in both positive ion mode (singly charged ion) and negative ion mode. Results revealed a molecular network consisting of nine hordatines, including hordatine B_1_ hexoside (**28**), hordatine B_2_ (**29**), hordatine B Glu (**30**), hordatine A Glu (**31**), hordatine C Glu (**32**), hordatine B_1_ (**33**), hordatine A_1_ (**34**), hordatine B (**35**), and hordatine A (**36**) (Figure 7A). In addition, four distinct node pairs were observed: network 14 (hordatines **30→37**), network 15 (hordatines **38→39**), network 18 (hordatines **32→39**), and network 40 (hordatines **40→33**) (Figure 7A). To elucidate how GNPS networks were generated and how structural assignments were made, hordatines B (**35**), B_1_ (**33**), and B_2_ (**29**) were selected as representative examples (Figure 7B). Hordatine B (**35**) exhibited characteristic fragment ions at *m*/*z* 451.1974 (C_24_H_27_O_5_N_4_), corresponding to the loss of one agmatine residue (130 Da) and at *m*/*z* 321.0757 (C_19_H_13_O_5_), corresponding to the loss of two agmatine residues (130 + 130 Da). Hordatines B_1_ (**33**) and B_2_ (**29**) showed similar fragmentation behavior: B_1_ (**33**) underwent sequential loss of one hydroxylated agmatine residue (146 Da) followed by one agmatine residue (130 Da), while B_2_ (**29**) lost two hydroxylated agmatine residues in sequence (146 + 146 Da). Both ultimately yielded fragment ions at *m*/*z* 321.0757 (C_19_H_13_O_5_) (Figure 7B). Overall, GNPS analysis in positive ion mode identified thirteen hordatines (**28**–**40**) distributed across five distinct molecular networks (Figures 7A).

**Figure 7:**
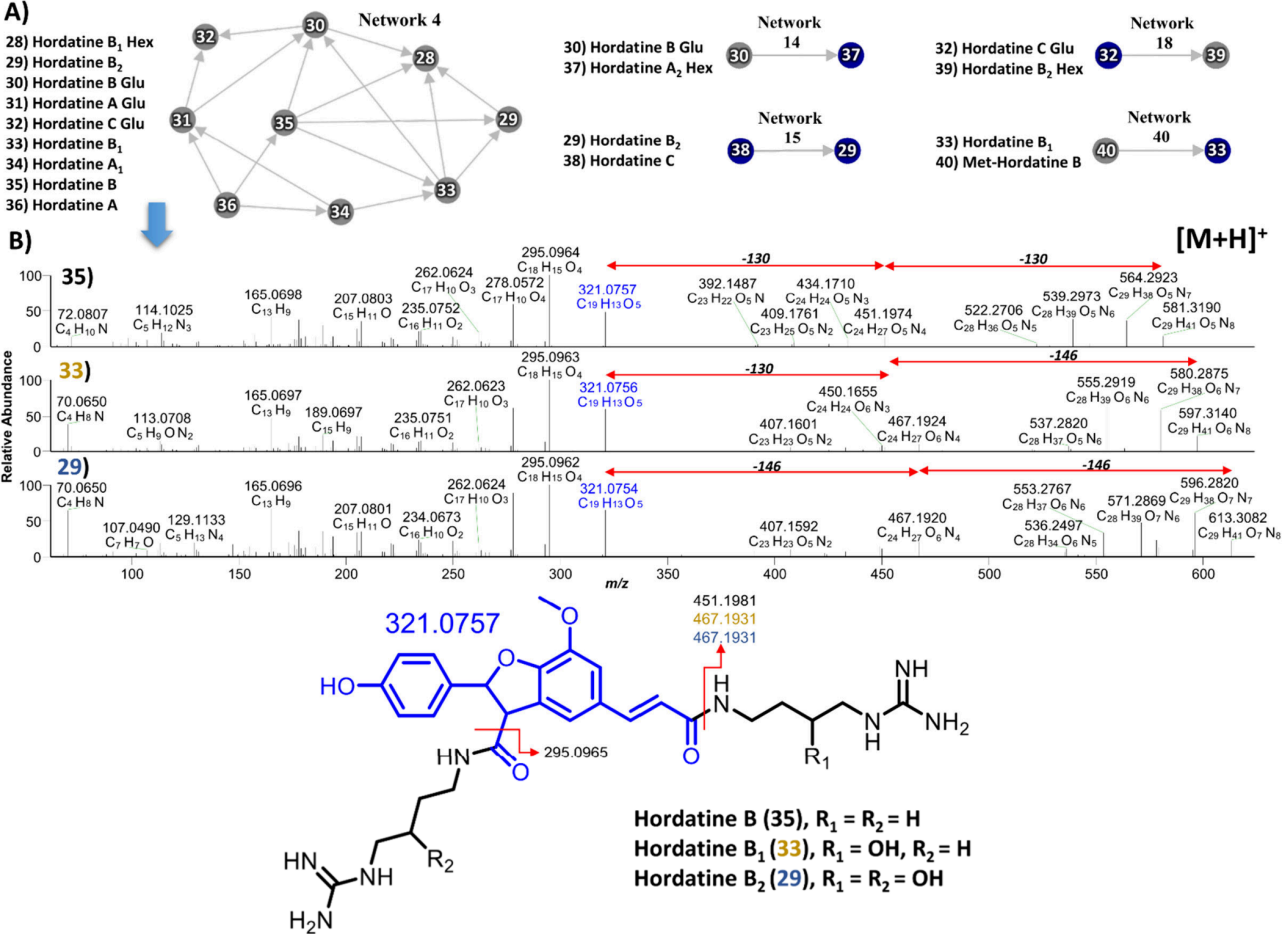
GNPS analysis of hordatine derivatives in whole grain barley in positive ion mode. (**A**) Five molecular networks of various hordatine derivatives (**28**–**40**), (**B**) MS^2^ spectra and proposed fragmentation patterns of hordatines B (**35**), B_1_ (**33**), and B_2_ (**29**).

In contrast, the negative ion mode revealed a more comprehensive molecular network of 18 hordatines (**27**–**39** and **41**–**45**; Figure 8A). It included not only the hordatines identified from five networks in positive ion mode except compound **40** but also three additional sugar-containing hordatines, hordatine A_1_ hexoside (**41**), C_1_ hexoside (**42**), and C_2_ hexoside (**43**), along with three additional hordatine aglycones, hordatine A_2_ (**27**), hordatine C_1_ (**44**), and C_2_ (**45**). This broader detection is potentially due to the presence of characteristic fragment ions with higher intensities in negative ion mode compared to those in positive ion mode (Figure 8A).

**Figure 8:**
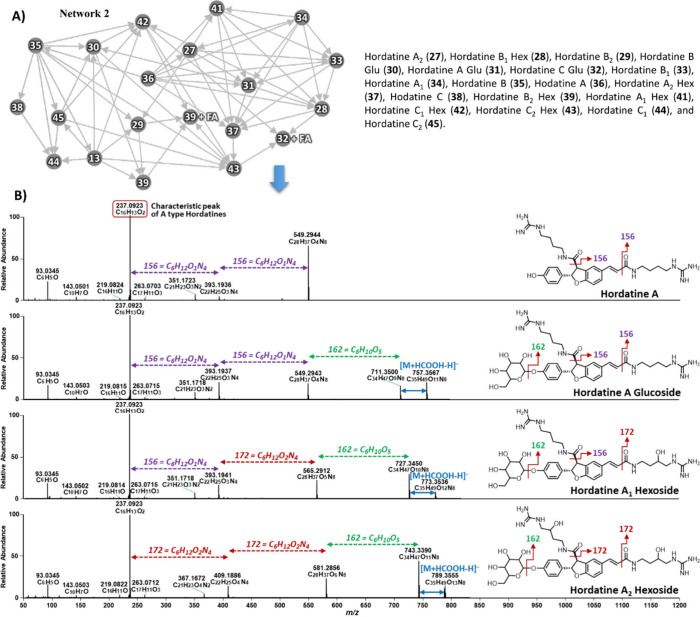
GNPS analysis of hordatine derivatives in whole grain barley in negative ion mode. (**A**) Molecular network of various hordatine derivatives (**27**–**39** and **41**–**45**), (**B**) MS^2^ spectra and proposed fragmentation patterns of hordatine A (**36**) and its glycosylated derivatives (hordatines **31**, **37**, and **41**).

To further elucidate how the GNPS network is generated and how the structures were elucidated, hordatine A (**36**), hordatine A glucoside (**31**), hordatine A_1_ hexoside (**41**), and hordatine A_2_ hexoside (**37**) were used as examples. In negative ion mode, these hordatines display specific neutral losses that are characteristic of their molecular structures. For example, hordatine A (**36**) produced a precursor ion at *m*/*z* 549.2944 [M – H]^−^ (C_28_H_37_O_4_N_8_), followed by fragment ions at *m*/*z* 393.1936 (C_22_H_25_O_3_N_4_) and at *m*/*z* 237.0923 (C_16_H_13_O_2_). These correspond to the losses of two agmatine molecules plus two carbonyl residues (156 Da × 2) (Figure 8A). In the case of glycosylated hordatines A (**31**), A_1_ (**41**), and A_2_ (**37**), although their base peak is associated with formic acid [M + HCOOH – H]^−^, their MS^2^ fragmentation initiates with the loss of a glucoside/hexoside (−162 Da) (Figure 8B). This is followed by two successive losses of 156 Da (C_6_H_12_O_1_N_4_; agmatine + carbonyl residue) for compound **31**, one loss of 156 Da and one loss of 172 Da (C_6_H_12_O_2_N_4_; hydroxyagmatine + carbonyl residue) for compound **51**, and two successive losses of 172 Da for compound **37**. These fragmentations are crucial for the formation of the observed molecular network. Interestingly, like positive ion mode, hordatines share a loss of 42 Da (CH_2_N_2_) in the negative ion mode, discerning the terminal part of the agmatine chain. Furthermore, all of these hordatines share a common characteristic peak for hordatine A at *m*/*z* 237.0923 with their corresponding network originating from different fragments of side chains (Figure 8B). Literature reports indicate that the *m*/*z* values of 237, 267, and 297 Da are key fragments in negative ion mode for recognizing the A, B, and C types of hordatines, respectively. Although the doubly charged ion in the positive ion mode is more favorable for the detection of hordatines,^8^ the negative ion mode may be preferable for GNPS networking. These fragment ions serve as molecular fingerprints, which allow for the identification of individual hordatines based on their unique mass-to-charge ratios.

### 3.9 Chemical Profile of Hordatines

Based on the structures of the 19 hordatines (**27**–**45**) identified by GNPS molecular networking, additional hordatines, such as methylated hordatines and di-, tri-, and tetra-glycosylated hordatines were manually searched from WG barley extract using LC/MS. Methylated hordatines B (**46**) and C (**47**) were found in the extract. Although previously reported di-, tri-, and tetra-glycosylated hordatines were detected based on their corresponding MS^1^, their quantities were insufficient for presence in either molecular network or the chemical fingerprint. The combination of GNPS molecular networking and knowledge-based information identified 21 major hordatines. These hordatines can be classified into seven groups, including hordatines A–C, hordatines A–C glucosides, hordatines A_1_–C_1_, hordatines A_1_–C_1_ hexosides, hordatines A_2_–C_2_, hordatines A_2_–C_2_ hexosides, and methylated hordatines A–C (Figure 9).

**Figure 9:**
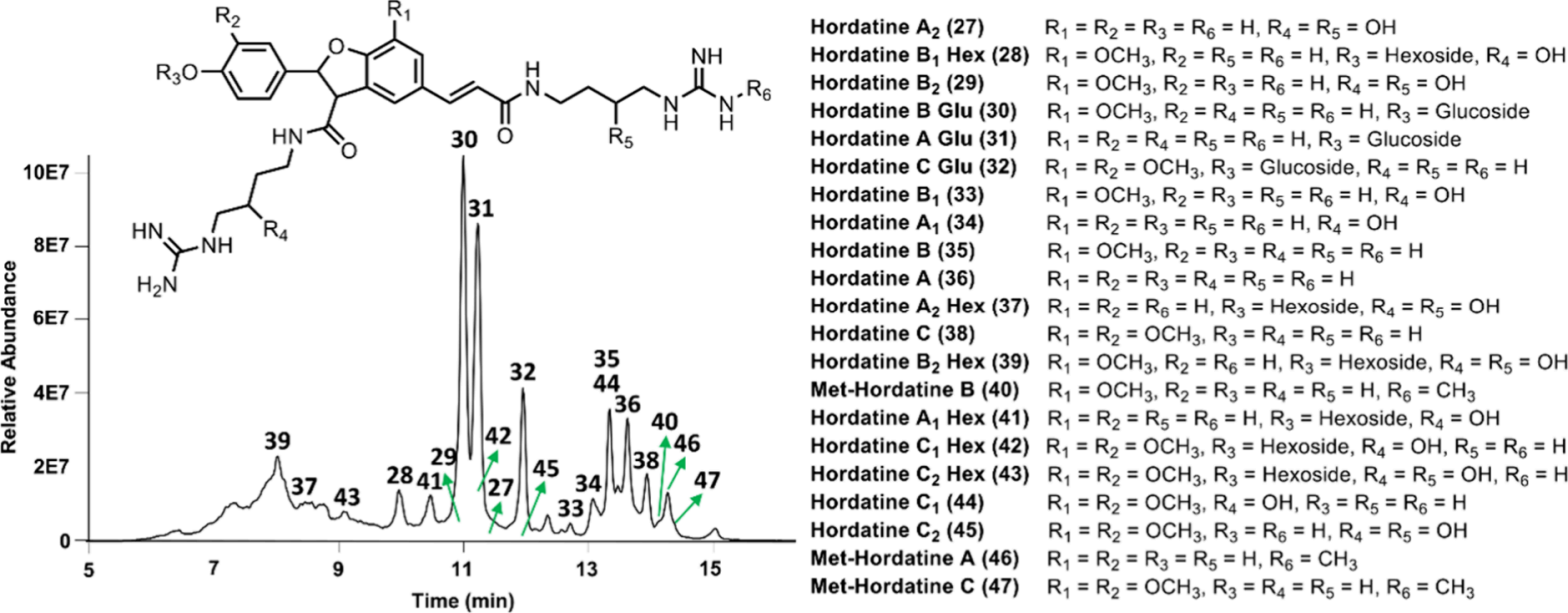
Chemical structures of hordatines identified in whole grain barley together with their chemical profile in the selected ion mode.

### 3.10 Quantification of Phenolamides in Barley-Based Products

To quantify phenolamides in commercial barley-based products, including alcoholic beers (*n* = 2 types), nonalcoholic beers (*n* = 6 types), bread (*n* = 1 type), flake (*n* = 1 type), flour (*n* = 4 types), hulled grain (*n* = 4 types), and pearl grain (*n* = 5 types), we developed a quantitative LC-HRMS method using previously synthesized and purified standards, such as Cou-Put, Cou-Agm, Fer-Agm, hordatines A–C, glycosylated hordatines A and B.^4^ In total, 50 phenolamides were identified, encompassing putrescine, spermidine, and agmatine conjugates as well as hordatines. Due to high variability observed in the levels of compounds **2a/2b**, **4**, **19**, **21**, and **24** across product types, quantitative analysis was performed on the remaining 44 phenolamides. Their total contents are presented in Figure 10A,B, with concentrations expressed in μg per 100 mL of beer or μg per 100 g of dry product. An example of the chemical profile of various barley-based products is shown in Figures S2A,B, while the quantification results have been summarized in Supplementary Tables S1–S5.

**Figure 10:**
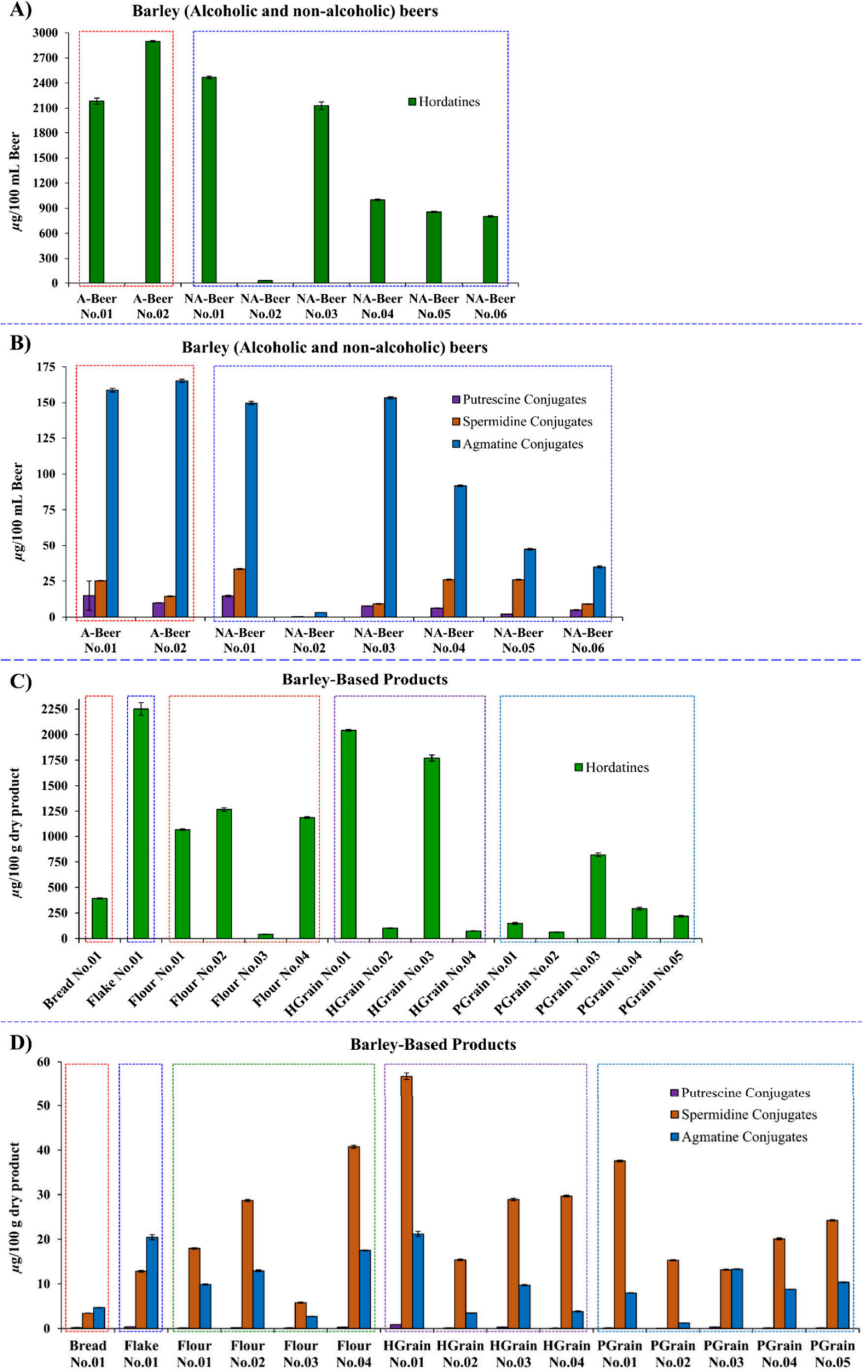
Total amount of various phenolamides, including putrescine, spermidine, and agmatine conjugates, as well as hordatines, in barley-based products; (**A**) Hordatines in alcoholic and nonalcoholic beers, (**B**) Putrescine, spermidine, and agmatine conjugates in alcoholic and nonalcoholic beers, (**C**) Hordatines in bread, flake, flour, hulled grain, and pearl grain, (**D**) Putrescine, spermidine, and agmatine conjugates in bread, flake, flour, hulled grain, and pearl grain.

Using a developed LC-HRMS method, 10 agmatine conjugates were quantified: Cou-Agm (**1**), Cou-OHAgm-Hex (**3**), CouAgm-Hex (**5**), Cou-OHAgm (**6**), FerAgm-Hex (**7**), Fer-OHAgm (**8**), SinAgm (**9**), Met-CouAgm (**10**), Fer-Agm (**11**), and Met-FerAgm (**12**). Compound **1** was used as the quantification standard for compounds **1**, **3**, **5**, **6**, and **10**, while compound **11** served as the standard for compounds **7**–**9**, **11**, and **12** (Tables S1 and S2). The total agmatine content across the barley-based products is listed in Figure 10. Our results showed that liquid products, except for Nonalcoholic Beer No.02, generally contained higher levels of agmatine conjugates compared to solid products (Figure S2B). However, the total quantity of agmatine conjugates varied among the solid products. Solid products such as bread and flour typically exhibited lower concentrations (0.14–1.48 and 0.01–5.06 μg/100 g dry product, respectively), with some individual compounds falling below the limit of quantification. In contrast, other barley products like Flake No.01 (0.19–7.35 μg/100 g dry product), Hulled Grain No.03 (0.13–4.87 μg/100 g dry product), and Pearl Grain No.03 (0.20–3.68 μg/100 g dry product) displayed higher variability. This suggested that the type of barley cultivar and the applied processing methods may affect the levels of these compounds.

For spermidine conjugates, 11 compounds were quantified: diCou-Spd (**13**), CouFer-Spd (**14**), FerCaf-Spd isomer 1 (**15a**), FerCaf-Spd isomer 2 (**15b**), diFer-Spd (**16**), diCaf-Spd (**17**), Caf-Spd (**18**), Fer-Spd (**20**), CouCaf-Spd isomer 1 (**22a**), CouCaf-Spd isomer 2 (**22b**), and Cou-Spd (**23**). Cou-Put (**25**) was used as the quantification standard. Table S3 presents the individual content of these conjugates, while their total concentrations are shown in Figure 10. Overall, our results showed a wide range of concentrations across various barley-based products, with significant differences between beers (ranging from 0.09 ± 0.01 to 33.49 ± 0.29 μg/100 mL) and dry products (ranging from 3.42 ± 0.03 to 56.76 ± 0.73 μg/100 g of dry product). Dry products, especially flours and hulled grains, generally displayed higher concentrations, with Hulled Grain No.01 showing the highest total content (56.76 ± 0.73 μg/100 g dry product). These findings highlight the importance of solid barley products in contributing to overall spermidine conjugates (Figure S2B). In contrast, Nonalcoholic Beer No.02, Bread No.01, and Flour No.04 displayed relatively low concentrations. The wide variation in both types and levels of spermidine conjugates across different products suggested that factors such as fermentation, production processes, and grain variety may influence their abundance.

Although the levels of putrescine conjugates (Cou-Put **25** and Fer-Put **26**), were generally low in barley products, both compounds were consistently detectable, with Fer-Put (**26**) present at higher concentrations than Cou-Put (**25**). Notably, their concentrations in beer products (0.24–1.05 μg/100 mL beer for compound **25** and 0.24–14.05 μg/100 mL beer for compound **26**) were higher compared to barely products in dried form, including flake, flour, bread, hulled grain, and pearl grain (0.05–0.27 μg/100 g product for compound **25** and 0.08–0.58 μg/100 g product for compound **26**) (Figure 10, Table S4).

In the case of hordatine derivatives, 21 compounds were quantified using LC-HRMS. Pure HA (**36**) was used as the quantification standard for HA_2_ (**27**), HA_1_ (**34**), HA (**36**), HA_2_G (**37**), HA_1_G (**41**), and Met-HA (**46**), while pure HB (**35**) served as the standard for HB_1_G (**28**), HB_2_ (**29**), HB_1_ (**33**), HB (**35**), HB_2_G (**39**), and Met-HB (**40**). Similarly, HC (**38**) was used to quantify HC (**38**), HCG (**32**), HC_1_ (**44**), HC_1_G (**42**), HC_2_ (**45**), HC_2_G (**43**), and Met-HC (**47**). Pure HAG (**31**) and HBG (**30**) were used as standards for their respective compounds (Table S1). Quantification results are summarized in Table S5, and the total hordatine content in barley-based products is presented in Figure 10. Among the tested samples, Alcoholic Beer No.02 exhibited the highest total hordatine content (2899.90 ± 8.52 μg/100 mL beer), followed by Nonalcoholic Beers No.01 and No.03 (2468.68 ± 15.35 and 2131.10 ± 43.39 μg/100 mL beer, respectively). Conversely, Nonalcoholic Beer No.02 showed considerably lower levels of most individual hordatines (Figures 10 and S2A). Although hordatine content varied among dry products, Flake No.01 (2251.12 ± 62.34 μg/100 g of dry product) and Hulled Grain No.01 (2041.29 ± 7.96 μg/100 g of dry product) displayed particularly high concentrations than more processed products like bread and pearl grains (Figure S2A). These observations indicated that less processed forms of barley, such as flake and hulled grains, retain higher levels of hordatines. Moreover, the variety of barley grain may also be a determinant factor. Consistent with our findings, Kohyama and Ono applied a pearling test across 10 barley cultivars, including six hulled and four hull-less varieties, and observed a significant reduction in HAG content following pearling. This result indicated that HAG is predominantly localized in the aleurone layer. They reported HAG levels in mature barley grains ranging from 57 to 140 mg·kg^–1^ dry weight when expressed as HA aglycone equivalents.^5^ Similarly, Pihlava et al. analyzed 208 commercial beers using HPLC-DAD with *p*-coumaric acid used as the standard and reported an average total hordatine content of 5.6 ± 3.1 mg.L^–1^ (expressed as *p*-coumaric acid equivalents, PCAE), with values ranging from 0 to 18.7 mg.L^–1^ PCAE.^14^

To the best of our knowledge, this is the first comprehensive study of WG barley phenolamides using GNPS molecular networking in combination with MS^2^ fragmentation patterns in both positive and negative ion modes to generate a detailed chemical fingerprint of these compounds. This integrative approach resulted in the identification of *p*-coumaroyl hydroxyagmatine derivatives, specifically *p*-coumaroyl hydroxydehydroagmatine (Cou-OHdhAgm) in two isomeric forms, *cis*-Cou-OHdhAgm (**2a**) and *trans*-Cou-OHdhAgm (**2b**). Our findings revealed that barley phenolamides are primarily composed of hordatines and their precursors (agmatine conjugates), followed by spermidine conjugates and, to a lower extent, putrescine conjugates. Of particular note is the first-time identification of sugar-containing spermidine conjugates (Fer-Spd-Hex **19**, diCaf-Spd-Hex **21**, and FerCaf-Spd-Hex **24**), as well as putrescine conjugates (Cou-Put **25** and Fer-Put **26**) in WG barley, expanding its known chemical diversity. In positive ion mode, hordatines generated both singly and doubly charged ions, with doubly charged species displaying a higher signal intensity. Meanwhile, their systematic fragmentation in negative ion mode provided valuable structural insights, enabling the construction of a detailed GNPS molecular network and allowing for a deeper exploration of hordatine diversity across barley varieties. For spermidine-based phenolamides, positive ion mode offered characteristic peaks, enabling rapid structural confirmation. Notably, the structures of 12 out of the 14 spermidine conjugates, including mono- (compounds **18**, **20**, and **23**), dihomo- (compounds **13**, **16**, and **17**), and diheteroconjugates (compounds **14**, **15a**/**15b**, and **22a**/**22b**), and a hexoside-containing dihomoconjugate (compound **21**), were fully elucidated for the first time. Quantification results across barley-based products indicated that beers were particularly rich in hordatines and their precursors. In contrast, hulled grains and flours exhibited higher concentrations of spermidine conjugates compared with beers, bread, and pearl grains. Among all products, barley bread contained the lowest levels of phenolamides, likely due to the effects of intensive processing.

While this study provides a comprehensive chemical and quantitative profile of barley phenolamides, it does have certain limitations. Notably, in vivo or functional bioactivity data are not included, which limits our ability to directly infer health outcomes. Additionally, limited availability of authentic standards for certain subclasses may affect the precision of quantification for less abundant or novel analogs.

Nevertheless, our findings strongly suggest that barley phenolamides may contribute to the nutritional and health-promoting properties of barley-based foods and beverages. However, these potential benefits are modulated by fermentation, food processing, and genotype-specific variation. These factors should be carefully considered in both future research and food formulation.

## Supplementary Material



## Notes

Standards employed for phenolamide quantification (Table S1), concentrations of agmatine, spermidine, and putrescine conjugates, as well as hordatines, across a range of barely products (Tables S2-S5), Calibration curves of authentic standards (Figure S1), ion chromatographic profiles of representative phenolamides detected in barley-based products (Figure S2) (PDF)

## Abbreviations

CE: Collision energies
ESI: Electrospray ionization
GNPS: Global Natural Products Social Molecular Networking
*N* ^10^3AP: *N* ^10^-(3-aminopropyl)
QC: Quality control
RSD: Relative Standard Deviation
UHPLC-HRESI-MS: Ultrahigh-pressure liquid chromatography coupled with high resolution electrospray mass spectrometry
