# Supplementary material for: Insights into Phenolamides in Whole Grain Barley: Chemical Profile and Their Levels in Barley-Based Products
Source: J Agric Food Chem. 2025 Aug 19;73(35):21905–19. doi: 10.1021/acs.jafc.5c04844 (PMC12412163; doi:10.1021/acs.jafc.5c04844)
Supplement: Supplementary file 1 [file jf5c04844_si_001.pdf]

Insights into phenolamides in whole grain barley: Chemical profile and their levels in barley-based products

Salar Hafez-Ghoran, Weixin Wang, Shengmin Sang*

Laboratory for Functional Foods and Human Health, Center for Excellence in Post-Harvest Technologies, North Carolina Agricultural and Technical State University, North Carolina Research Campus, 500 Laureate Way, Kannapolis, North Carolina 28081, United States.

***Correspondence:**
Dr. Shengmin Sang
Phone: 704-250-5710
Email addresses: ssang@ncat.edu

Emails and ORCIDs:
Salar Hafez-Ghoran (shafezghoran@ncat.edu & 0000-0001-5495-5556)
Weixin Wang (wwang@ncat.edu)
Shengmin Sang (ssang@ncat.edu & 0000-0002-5005-3616)

Contents	Page
**Table S1**. Standards used for quantification of barley phenolamides.	3
**Table S2.** The abundances of agmatine conjugates, including Cou-Agm (**1**), Cou-OHAgm-Hex (**3**), CouAgm-Hex (**5**), Cou-OHAgm (**6**), FerAgm-Hex (**7**), Fer-OHAgm (**8**), SinAgm (**9**), Met-CouAgm (**10**), Fer-Agm (**11**), and Met-FerAgm (**12**), in various barley products using **1** and **11** as standards for quantification. The data are based on µg/100 mL beer (for beers) and µg/100 g dry product (for other products).	4
**Table S3.** The abundances of spermidine conjugates, including diCou-Spd (**13**), CouFer-Spd (**14**), FerCaf-Spd isomer 1 (**15a**), FerCaf-Spd isomer 2 (**15b**), diFer-Spd (**16**), diCaf-Spd (**17**), Caf-Spd (**18**), Fer-Spd (**20**), CouCaf-Spd isomer 1 (**22a**), CouCaf-Spd isomer 2 (**22b**), and Cou-Spd (**23**), in various barley-based products using Cou-Put (**25**) as standard for quantification. The data are based on µg/100 mL beer (for beers) and µg/100 g dry product (for other products).	5
**Table S4**. The abundances of putrescine conjugates, including Cou-Put (**25**) and Fer-Put (**26**) in various barley-based products using Cou-Put (**25**) as standard for quantification. The data are based on µg/100 mL beer (for beers) and µg/100 g dry product (for other products).	6
**Table S5.** The abundances of hrodatines in various barley products using HA, HAG, HB, HBG, and HC as standard for quantification. The data are based on µg/100 mL beer (for beers) and µg/100 g dry product (for other products).	7
**Figure S1**. The standard curves of Hordatines A–C, glycosylated hordatines A and B, Cou-Agm, Fer-Agm, and Cou-Put.	8
**Figure S2**. Chemical profile of phenolamides in barley-based products (selected ions with fixed scale); **A**) Agmatine and spermidine conjugates, **B**) Hordatines.	9

Table S1. Standards used for quantification of barley phenolamides.

**Standard**	***Quantification***
**Hordatine A**	*HA*, HA_1, HA_1G, HA_2, HA_2G, *Met-HA*
**Hordatine B**	*HB*, HB_1, HB_2, HB_1G, HB_2G, *Met-HB*
**Hordatine C**	*HC, HCG*, HC_1, HC_1G, HC_2, HC_2G, *Met-HC*
**Hordatine A Glu**	*HAG*
**Hordatine B Glu**	*HBG*
**Cou-Agm**	*CouAgm, CouAgm-Hex, Cou-OHAgm, Cou-OHAgm-Hex, Met-CouAgm*
**Fer-Agm**	*Fer-Agm, FerAgm-Hex, Fer-OHAgm, Met-FerAgm, Sin-Agm*
**Cou-Put**	*Cou-Put, Fer-Put, Cou-Spd, Caf-Spd, Fer-Spd, diCou-Spd, diCaf-Spd, diFer-Spd, FerCaf-Spd (isomer 1), FerCaf-Spd (isomer 2), CouCaf-Spd (isomer 1), CouCaf-Spd (isomer 2), and CouFer-Spd.*

Table S2. The abundances of agmatine conjugates, including Cou-Agm (**1**), Cou-OHAgm-Hex (**3**), CouAgm-Hex (**5**), Cou-OHAgm (**6**), FerAgm-Hex (**7**), Fer-OHAgm (**8**), SinAgm (**9**), Met-CouAgm (**10**), Fer-Agm (**11**), and Met-FerAgm (**12**), in various barley products using **1** and **11** as standards for quantification. The data are based on µg/100 mL beer (for beers) and µg/100 g dry product (for other products).

	**Agmatine Conjugates***										**Total**
	**Cou-Agm[a]**	CouAgm-Hex	Cou-OHAgm	Cou-OHAgm-Hex	Met-CouAgm	**Fer-Agm[a]**	FerAgm-Hex	Fer-OHAgm	Met-FerAgm	Sin-Agm	
**Barley Products**	**µg/100 mL beer**										
Alcoholic Beer No.01	57.37 ± 0.93	1.49 ± 0.10	38.32 ± 0.25	0.62 ± 0.01	1.61 ± 0.04	30.10 ± 0.76	< LOQ[b]	27.29 ± 0.46	1.28 ± 0.00	0.74 ± 0.01	158.82 ± 1.31
Alcoholic Beer No.02	16.11 ± 0.12	1.32 ± 0.04	55.41 ± 1.05	1.15 ± 0.03	0.99 ± 0.04	33.78 ± 0.16	–	54.32 ± 0.17	1.05 ± 0.01	1.14 ± 0.03	165.27 ± 1.08
Non-alcoholic Beer No.01	36.29 ± 0.71	2.10 ± 0.03	40.07 ± 0.15	0.76 ± 0.00	1.66 ± 0.01	34.39 ± 1.00	< LOQ[b]	32.03 ± 0.51	1.53 ± 0.04	0.79 ± 0.02	149.62 ± 1.34
Non-alcoholic Beer No.02	0.49 ± 0.00	< LOQ[b]	1.22 ± 0.02	< LOQ[b]	< LOQ	0.49 ± 0.01	< LOQ[b]	1.01 ± 0.01	< LOQ[b]	< LOQ[b]	3.21 ± 0.025
Non-alcoholic Beer No.03	35.97 ± 0.28	0.97 ± 0.02	42.97 ± 0.33	0.98 ± 0.04	1.06 ± 0.03	29.26 ± 0.60	0.47 ± 0.02	39.92 ± 0.16	0.96 ± 0.06	0.87 ± 0.01	153.43 ± 0.76
Non-alcoholic Beer No.04	37.91 ± 0.18	0.73 ± 0.02	16.99 ± 0.40	0.18 ± 0.01	1.20 ± 0.05	18.50 ± 0.30	0.35 ± 0.00	14.64 ± 0.01	0.99 ± 0.03	0.36 ± 0.00	91.85 ± 0.54
Non-alcoholic Beer No.05	8.40 ± 0.38	0.22 ± 0.02	12.29 ± 0.12	< LOQ[b]	0.69 ± 0.03	13.40 ± 0.30	< LOQ[b]	11.02 ± 0.06	0.82 ± 0.02	0.41 ± 0.00	47.25 ± 0.50
Non-alcoholic Beer No.06	10.29 ± 0.55	0.89 ± 0.05	7.67 ± 0.48	0.34 ± 0.00	0.55 ± 0.00	8.64 ± 0.06	0.37 ± 0.00	5.67 ± 0.09	0.38 ± 0.00	0.18 ± 0.02	34.98 ± 0.74
	**Cou-Agm[a]**	CouAgm-Hex	Cou-OHAgm	Cou-OHAgm-Hex	Met-CouAgm	**Fer-Agm[a]**	FerAgm-Hex	Fer-OHAgm	Met-FerAgm	Sin-Agm	**Total**
**Barley Products**	**µg/100 g dry product**										
Bread No.01	0.45 ± 0.00	0.16 ± 0.00	1.20 ± 0.00	< LOQ[b]	0.14 ± 0.00	0.79 ± 0.01	< LOQ[b]	1.48 ± 0.02	0.21 ± 0.01	0.24 ± 0.01	4.67 ± 0.03
Flake No.01	7.35 ± 0.53	2.33 ± 0.08	2.71 ± 0.12	0.47 ± 0.01	0.67 ± 0.00	4.52 ± 0.11	0.79 ± 0.00	1.02 ± 0.01	0.58 ± 0.01	< LOQ[b]	20.44 ± 0.56
Flour No.01	1.79 ± 0.06	0.55 ± 0.00	0.99 ± 0.06	0.22 ± 0.00	0.51 ± 0.00	1.86 ± 0.00	0.26 ± 0.01	2.42 ± 0.07	0.73 ± 0.01	0.45 ± 0.01	9.87 ± 0.11
Flour No.02	3.29 ± 0.06	0.60 ± 0.01	2.11 ± 0.13	0.26 ± 0.00	0.38 ± 0.01	1.43 ± 0.09	0.26 ± 0.02	3.72 ± 0.09	0.54 ± 0.01	0.32 ± 0.02	12.91 ± 0.19
Flour No.03	0.56 ± 0.00	< LOQ[b]	1.03 ± 0.02	< LOQ[b]	< LOQ[b]	0.61 ± 0.00	< LOQ[b]	0.50 ± 0.01	< LOQ[b]	< LOQ[b]	2.70 ± 0.02
Flour No.04	5.06 ± 0.03	0.01 ± 0.01	2.09 ± 0.03	0.46 ± 0.00	1.05 ± 0.03	2.86 ± 0.00	0.38 ± 0.02	3.86 ± 0.07	1.29 ± 0.02	0.43 ± 0.00	17.49 ± 0.10
Hulled Grain No.01	3.14 ± 0.32	1.43 ± 0.03	2.73 ± 0.45	0.88 ± 0.02	1.31 ± 0.00	3.08 ± 0.08	0.44 ± 0.02	6.20 ± 0.09	1.32 ± 0.00	0.68 ± 0.00	21.21 ± 0.57
Hulled Grain No.02	0.58 ± 0.01	< LOQ[b]	0.79 ± 0.02	< LOQ[b]	< LOQ[b]	0.56 ± 0.02	< LOQ[b]	1.20 ± 0.03	0.16 ± 0.00	0.17 ± 0.00	3.46 ± 0.04
Hulled Grain No.03	4.87 ± 0.04	0.93 ± 0.02	1.15 ± 0.04	0.21 ± 0.01	0.43 ± 0.01	0.32 ± 0.10	0.33 ± 0.01	0.76 ± 0.02	0.60 ± 0.03	0.13 ± 0.01	9.73 ± 0.13
Hulled Grain No.04	0.30 ± 0.01	< LOQ[b]	1.08 ± 0.02	< LOQ[b]	< LOQ[b]	0.32 ± 0.15	< LOQ[b]	1.98 ± 0.01	< LOQ[b]	0.13 ± 0.00	3.81 ± 0.15
Pearl Grain No.01	1.01 ± 0.01	0.22 ± 0.01	1.92 ± 0.04	0.65 ± 0.01	0.09 ± 0.00	0.78 ± 0.02	0.13 ± 0.00	2.74 ± 0.04	0.15 ± 0.00	0.26 ± 0.01	7.95 ± 0.05
Pearl Grain No.02	0.16 ± 0.00	< LOQ[b]	0.13 ± 0.01	< LOQ[b]	< LOQ[b]	0.54 ± 0.01	< LOQ[b]	0.40 ± 0.01	< LOQ[b]	< LOQ[b]	1.23 ± 0.02
Pearl Grain No.03	3.17 ± 0.09	0.59 ± 0.00	3.39 ± 0.01	0.48 ± 0.00	0.35 ± 0.00	0.68 ± 0.02	0.29 ± 0.00	3.68 ± 0.04	0.44 ± 0.02	0.20 ± 0.01	13.27 ± 0.10
Pearl Grain No.04	2.52 ± 0.02	0.35 ± 0.01	2.22 ± 0.02	0.24 ± 0.01	0.32 ± 0.01	1.51 ± 0.01	0.12 ± 0.00	0.97 ± 0.01	0.36 ± 0.00	0.17 ± 0.01	8.78 ± 0.04
Pearl Grain No.05	2.14 ± 0.02	0.37 ± 0.00	3.67 ± 0.05	0.31 ± 0.01	0.21 ± 0.01	1.60 ± 0.05	0.18 ± 0.02	1.47 ± 0.02	0.27 ± 0.01	0.15 ± 0.00	10.37 ± 0.08

[a]Standard for quantification, [b]LOQ: Lower of quantification.

*The quantification of barley phenolamides was analyzed in two independent samples.

Table S3. The abundances of spermidine conjugates, including diCou-Spd (**13**), CouFer-Spd (**14**), FerCaf-Spd isomer 1 (**15a**), FerCaf-Spd isomer 2 (**15b**), diFer-Spd (**16**), diCaf-Spd (**17**), Caf-Spd (**18**), Fer-Spd (**20**), CouCaf-Spd isomer 1 (**22a**), CouCaf-Spd isomer 2 (**22b**), and Cou-Spd (**23**), in various barley-based products using Cou-Put (**25**) as standard for quantification. The data are based on µg/100 mL beer (for beers) and µg/100 g dry product (for other products).

	**Spermidine Conjugates (Cou-Put[a])***											**Total**
	Cou-Spd	Caf-Spd	Fer-Spd	diCou-Spd	diCaf-Spd	diFer-Spd	FerCaf-Spd1	FerCaf-Spd2	CouCaf-Spd1	CouCaf-Spd2	CouFer-Spd	
**Barley Products**	**µg/100 mL beer**											
Alcoholic Beer No.01	0.64 ± 0.06	0.15 ± 0.01	2.09 ± 0.05	1.92 ± 0.01	0.91 ± 0.01	9.80 ± 0.17	1.04 ± 0.05	1.46 ± 0.02	1.20 ± 0.00	2.53 ± 0.04	3.55 ± 0.05	25.29 ± 0.21
Alcoholic Beer No.02	0.94 ± 0.01	0.47 ± 0.02	4.07 ± 0.24	0.57 ± 0.02	0.41 ± 0.02	4.29 ± 0.02	0.58 ± 0.00	0.96 ± 0.01	0.45 ± 0.03	0.64 ± 0.00	1.14 ± 0.03	14.52 ± 0.25
Non-alcoholic Beer No.01	0.73 ± 0.04	0.19 ± 0.01	2.29 ± 0.06	2.72 ± 0.11	2.17 ± 0.03	10.72 ± 0.12	1.74 ± 0.05	2.03 ± 0.03	2.10 ± 0.07	4.05 ± 0.10	4.75 ± 0.05	33.49 ± 0.29
Non-alcoholic Beer No.02	< LOQ[b]	< LOQ[b]	0.09 ± 0.01	< LOQ[b]	< LOQ[b]	< LOQ[b]	–	< LOQ[b]	–	< LOQ[b]	< LOQ[b]	0.09 ± 0.01
Non-alcoholic Beer No.03	0.91 ± 0.02	0.25 ± 0.01	2.20 ± 0.08	0.55 ± 0.03	0.06 ± 0.02	2.50 ± 0.13	0.25 ± 0.00	0.63 ± 0.00	0.29 ± 0.02	0.56 ± 0.01	1.04 ± 0.00	9.24 ± 0.16
Non-alcoholic Beer No.04	0.47 ± 0.02	0.16 ± 0.00	1.31 ± 0.02	1.97 ± 0.05	4.32 ± 0.31	5.59 ± 0.11	1.62 ± 0.03	1.77 ± 0.00	2.32 ± 0.05	3.89 ± 0.02	2.70 ± 0.04	26.12 ± 0.34
Non-alcoholic Beer No.05	0.55 ± 0.00	0.16 ± 0.01	1.71 ± 0.05	2.22 ± 0.09	3.08 ± 0.01	6.31 ± 0.08	1.41 ± 0.01	1.60 ± 0.02	2.25 ± 0.18	3.59 ± 0.16	3.17 ± 0.04	26.05 ± 0.28
Non-alcoholic Beer No.06	0.34 ± 0.03	0.10 ± 0.00	0.63 ± 0.00	0.95 ± 0.00	0.75 ± 0.00	2.54 ± 0.17	0.52 ± 0.02	0.52 ± 0.01	0.64 ± 0.02	1.00 ± 0.04	1.14 ± 0.06	9.13 ± 0.19
	Cou-Spd	Caf-Spd	Fer-Spd	diCou-Spd	diCaf-Spd	diFer-Spd	FerCaf-Spd1	FerCaf-Spd2	CouCaf-Spd1	CouCaf-Spd2	CouFer-Spd	**Total**
**Barley Products**	**µg/100 g dry product**											
Bread No.01	0.09 ± 0.00	0.09 ± 0.00	0.39 ± 0.01	0.22 ± 0.00	0.10 ± 0.00	0.58 ± 0.02	0.35 ± 0.01	0.30 ± 0.01	0.42 ± 0.01	0.26 ± 0.00	0.62 ± 0.02	3.42 ± 0.03
Flake No.01	1.01 ± 0.03	0.29 ± 0.00	4.19 ± 0.17	0.36 ± 0.00	1.10 ± 0.05	1.96 ± 0.06	1.02 ± 0.05	0.91 ± 0.04	0.55 ± 0.00	0.53 ± 0.02	0.90 ± 0.03	12.82 ± 0.20
Flour No.01	2.24 ± 0.08	2.32 ± 0.04	5.89 ± 0.07	0.48 ± 0.02	2.67 ± 0.07	1.00 ± 0.01	0.55 ± 0.01	0.66 ± 0.02	0.74 ± 0.00	0.81 ± 0.02	0.57 ± 0.00	17.93 ± 0.14
Flour No.02	2.46 ± 0.11	1.64 ± 0.08	10.25 ± 0.13	0.84 ± 0.07	3.75 ± 0.04	2.45 ± 0.07	1.64 ± 0.00	1.67 ± 0.06	1.32 ± 0.02	1.21 ± 0.06	1.46 ± 0.04	28.69 ± 0.24
Flour No.03	0.75 ± 0.01	0.50 ± 0.03	1.60 ± 0.06	0.22 ± 0.01	0.21 ± 0.00	0.71 ± 0.09	0.30 ± 0.00	0.43 ± 0.00	0.26 ± 0.01	0.31 ± 0.02	0.49 ± 0.00	5.78 ± 0.12
Flour No.04	4.18 ± 0.14	2.90 ± 0.00	17.24 ± 0.24	0.98 ± 0.04	2.57 ± 0.05	4.33 ± 0.01	1.60 ± 0.07	2.31 ± 0.02	0.89 ± 0.03	1.20 ± 0.04	2.54 ± 0.10	40.74 ± 0.31
Hulled Grain No.01	3.50 ± 0.01	6.79 ± 0.02	18.85 ± 0.66	0.62 ± 0.02	15.19 ± 0.30	2.31 ± 0.04	1.32 ± 0.01	2.89 ± 0.08	1.89 ± 0.03	2.14 ± 0.03	1.26 ± 0.01	56.76 ± 0.73
Hulled Grain No.02	1.73 ± 0.12	0.79 ± 0.01	4.32 ± 0.01	0.50 ± 0.02	1.32 ± 0.05	2.51 ± 0.01	0.77 ± 0.01	1.12 ± 0.04	0.51 ± 0.02	0.61 ± 0.01	1.20 ± 0.01	15.38 ± 0.14
Hulled Grain No.03	1.10 ± 0.02	0.20 ± 0.00	2.96 ± 0.17	2.49 ± 0.00	6.96 ± 0.10	3.32 ± 0.03	1.82 ± 0.03	2.04 ± 0.02	1.94 ± 0.03	3.52 ± 0.13	2.58 ± 0.10	28.93 ± 0.26
Hulled Grain No.04	3.21 ± 0.01	4.86 ± 0.05	6.26 ± 0.15	0.81 ± 0.00	6.10 ± 0.05	1.62 ± 0.01	1.08 ± 0.03	1.62 ± 0.02	1.42 ± 0.05	1.54 ± 0.06	1.15 ± 0.05	29.67 ± 0.19
Pearl Grain No.01	0.43 ± 0.00	0.31 ± 0.00	0.50 ± 0.00	3.06 ± 0.01	10.01 ± 0.03	4.92 ± 0.02	2.58 ± 0.06	2.54 ± 0.08	3.60 ± 0.19	5.41 ± 0.01	4.18 ± 0.00	37.54 ± 0.22
Pearl Grain No.02	1.58 ± 0.05	1.81 ± 0.01	2.20 ± 0.02	0.46 ± 0.01	4.21 ± 0.03	0.96 ± 0.02	0.70 ± 0.03	0.88 ± 0.00	0.94 ± 0.07	1.00 ± 0.11	0.55 ± 0.00	15.29 ± 0.15
Pearl Grain No.03	0.35 ± 0.00	0.12 ± 0.00	0.64 ± 0.00	0.79 ± 0.02	2.95 ± 0.05	2.74 ± 0.06	1.06 ± 0.00	1.05 ± 0.03	0.78 ± 0.04	1.22 ± 0.04	1.46 ± 0.04	13.16 ± 0.11
Pearl Grain No.04	2.39 ± 0.01	1.53 ± 0.06	6.64 ± 0.18	0.70 ± 0.02	1.36 ± 0.01	2.49 ± 0.05	0.96 ± 0.00	1.15 ± 0.07	0.73 ± 0.00	0.71 ± 0.02	1.44 ± 0.02	20.10 ± 0.21
Pearl Grain No.05	3.44 ± 0.09	2.65 ± 0.05	8.82 ± 0.05	0.80 ± 0.04	1.62 ± 0.02	2.00 ± 0.08	0.88 ± 0.02	1.21 ± 0.03	0.70 ± 0.00	0.76 ± 0.01	1.36 ± 0.01	24.24 ± 0.15

[a]Standard for quantification, [b]LOQ: Lower of quantification.

*The quantification of barley phenolamides was analyzed in two independent samples.

Table S4. The abundances of putrescine conjugates, including Cou-Put (**25**) and Fer-Put (**26**) in various barley products using Cou-Put (**25**) as standard for quantification. The data are based on µg/100 mL beer (for beers) and µg/100 g dry product (for other products).

Putrescine Conjugates*			Total
	**Cou-Put[a]**	Fer-Put	
**Barley Products**	**µg/100 mL beer**		
Alcoholic Beer No.01	1.05 ± 0.07	13.95 ± 0.15	15.00 ± 0.17
Alcoholic Beer No.02	0.43 ± 0.00	9.42 ± 0.08	9.85 ± 0.08
Non-alcoholic Beer No.01	0.65 ± 0.00	14.05 ± 0.65	14.70 ± 0.65
Non-alcoholic Beer No.02	< LOQ[b]	0.24 ± 0.00	0.24 ± 0.00
Non-alcoholic Beer No.03	0.60 ± 0.01	7.10 ± 0.09	7.70 ± 0.09
Non-alcoholic Beer No.04	0.67 ± 0.00	5.57 ± 0.06	6.24 ± 0.06
Non-alcoholic Beer No.05	0.24 ± 0.00	1.80 ± 0.01	2.04 ± 0.01
Non-alcoholic Beer No.06	0.52 ± 0.01	4.46 ± 0.27	4.98 ± 0.27
	**Cou-Put[a]**	Fer-Put	**Total**
**Barley Products**	**µg/100 g dry product**		
Bread No.01	0.08 ± 0.00	0.14 ± 0.01	0.22 ± 0.01
Flake No.01	0.16 ± 0.01	0.20 ± 0.00	0.36 ± 0.01
Flour No.01	< LOQ[b]	0.11 ± 0.00	0.11 ± 0.00
Flour No.02	< LOQ[b]	0.18 ± 0.00	0.18 ± 0.00
Flour No.03	< LOQ[b]	0.13 ± 0.01	0.13 ± 0.01
Flour No.04	0.07 ± 0.00	0.22 ± 0.01	0.29 ± 0.01
Hulled Grain No.01	0.27 ± 0.01	0.58 ± 0.02	0.85 ± 0.02
Hulled Grain No.02	0.05 ± 0.00	0.08 ± 0.00	0.13 ± 0.00
Hulled Grain No.03	0.09 ± 0.02	0.21 ± 0.00	0.30 ± 0.02
Hulled Grain No.04	–	0.09 ± 0.03	0.09 ± 0.03
Pearl Grain No.01	< LOQ[b]	0.12 ± 0.01	0.12 ± 0.01
Pearl Grain No.02	–	0.08 ± 0.00	0.08 ± 0.00
Pearl Grain No.03	0.12 ± 0.00	0.21 ± 0.01	0.33 ± 0.01
Pearl Grain No.04	–	0.10 ± 0.00	0.10 ± 0.00
Pearl Grain No.05	< LOQ[b]	0.13 ± 0.00	0.13 ± 0.00

[a]Standard for quantification, [b]LOQ: Lower of quantification.

*The quantification of barley phenolamides was analyzed in two independent samples.

Table S5. The abundances of hrodatines in various barley products using HA, HAG, HB, HBG, and HC as standard for quantification.

Barley Products	HA[a]	HAG[a]	HB[a]	HBG[a]	HC[a]	HCG	HA1	HA1G	HB1	HB1G	HC1	HC1G	HA2	HA2G	HB2	HB2G	HC2	HC2G	Met-HA	Met-HB	Met-HC	Total
	**Hordatines***																					
	**µg/100 mL beer**																					
Alcoholic Beer No.01	844.32 ± 29.97	89.19 ± 9.20	612.87 ± 17.30	284.88 ± 7.85	215.47 ± 2.15	0.38 ± 0.01	24.37 ± 0.72	3.46 ± 0.02	8.13 ± 0.19	13.00 ± 0.09	14.15 ± 0.05	0.40 ± 0.03	13.50 ± 0.05	2.35 ± 0.11	28.35 ± 0.91	7.78 ± 0.38	10.87 ± 0.12	–	2.43 ± 0.00	1.87 ± 0.07	6.94 ± 0.03	2184.71 ± 36.74
Alcoholic Beer No.02	1113.61 ± 5.03	84.33 ± 1.43	930.11 ± 0.80	279.13 ± 6.25	254.95 ± 2.21	0.30 ± 0.02	29.44 ± 0.00	3.18 ± 0.12	13.04 ± 0.08	15.94 ± 0.57	24.20 ± 0.33	1.19 ± 0.11	25.71 ± 0.05	2.93 ± 0.12	70.34 ± 0.07	15.93 ± 0.34	26.64 ± 0.11	–	2.35 ± 0.04	1.59 ± 0.02	4.99 ± 0.04	2899.90 ± 8.52
Non-alcoholic Beer No.01	959.13 ± 1.39	78.06 ± 13.07	736.35 ± 7.42	320.17 ± 2.74	257.30 ± 0.04	0.33 ± 0.00	23.22 ± 0.06	2.96 ± 0.14	8.21 ± 0.07	12.42 ± 0.37	13.67 ± 0.15	0.29 ± 0.03	9.23 ± 0.40	1.55 ± 0.09	19.19 ± 0.25	5.54 ± 0.09	8.32 ± 0.07	–	2.64 ± 0.05	2.31 ± 0.03	7.79 ± 0.01	2468.68 ± 15.35
Non-alcoholic Beer No.02	3.52 ± 0.03	8.85 ± 0.04	2.94 ± 0.03	10.76 ± 0.20	0.71 ± 0.00	1.29 ± 0.02	0.22 ± 0.03	0.22 ± 0.03	< LOQ[b]	0.58 ± 0.01	< LOQ[b]	0.26 ± 0.01	0.27 ± 0.01	0.16 ± 0.01	0.53 ± 0.01	0.49 ± 0.01	< LOQ[b]	0.17 ± 0.01	< LOQ[b]	< LOQ[b]	–	30.97 ± 0.22
Non-alcoholic Beer No.03	576.35 ± 41.43	378.79 ± 2.39	507.58 ± 12.55	413.62 ± 0.06	76.43 ± 0.91	52.28 ± 0.84	–	4.30 ± 0.20	9.81 ± 0.38	14.96 ± 0.41	5.07 ± 0.13	8.68 ± 0.47	12.71 ± 0.14	2.62 ± 0.05	41.97 ± 0.67	10.56 ± 0.01	5.08 ± 0.03	6.13 ± 0.15	1.58 ± 0.07	1.18 ± 0.01	1.40 ± 0.04	2131.10 ± 43.39
Non-alcoholic Beer No.04	154.54 ± 0.39	185.86 ± 3.55	174.30 ± 2.99	279.21 ± 6.80	133.44 ± 0.81	8.87 ± 0.84	11.81 ± 0.21	4.56 ± 0.10	4.16 ± 0.10	9.20 ± 0.35	6.79 ± 0.03	1.03 ± 0.03	4.28 ± 0.00	1.07 ± 0.05	8.78 ± 0.45	1.50 ± 0.11	2.83 ± 0.10	0.29 ± 0.02	0.76 ± 0.00	1.06 ± 0.02	4.89 ± 0.10	999.23 ± 8.35
Non-alcoholic Beer No.05	198.17 ± 1.11	44.62 ± 0.53	269.57 ± 3.84	136.37 ± 3.97	79.20 ± 1.44	51.10 ± 0.60	6.85 ± 0.04	1.11 ± 0.08	5.85 ± 0.39	3.23 ± 0.11	4.22 ± 0.01	3.91 ± 0.01	10.68 ± 0.18	0.73 ± 0.05	31.95 ± 0.32	2.12 ± 0.09	4.68 ± 0.00	3.19 ± 0.05	0.77 ± 0.04	1.03 ± 0.02	1.66 ± 0.03	854.27 ± 6.77
Non-alcoholic Beer No.06	102.20 ± 1.21	273.28 ± 6.93	83.37 ± 0.68	260.02 ± 5.73	20.55 ± 0.01	27.57 ± 0.09	3.46 ± 0.01	4.34 ± 0.07	1.97 ± 0.12	9.12 ± 0.35	0.96 ± 0.04	2.51 ± 0.06	2.67 ± 0.13	1.83 ± 0.06	5.73 ± 0.16	4.06 ± 0.01	0.91 ± 0.02	1.39 ± 0.05	0.52 ± 0.01	0.42 ± 0.01	0.59 ± 0.02	803.00 ± 8.94
	HA[a]	HAG[a]	HB[a]	HBG[a]	HC[a]	HCG	HA1	HA1G	HB1	HB1G	HC1	HC1G	HA2	HA2G	HB2	HB2G	HC2	HC2G	Met-HA	Met-HB	Met-HC	Total
**Barley Products**	**µg/100 g dry product**																					
Bread No.01	26.10 ± 0.57	49.30 ± 0.77	39.54 ± 2.28	183.45 ± 3.52	11.82 ± 0.51	25.49 ± 0.75	3.54 ± 0.15	4.02 ± 0.10	4.72 ± 0.12	10.15 ± 0.08	0.83 ± 0.02	2.42 ± 0.17	5.96 ± 0.08	6.30 ± 0.28	7.21 ± 0.01	11.77 ± 0.44	0.91 ± 0.02	2.21 ± 0.13	0.18 ± 0.00	0.22 ± 0.11	0.55 ± 0.02	392.13 ± 6.66
Flake No.01	58.09 ± 1.76	740.62 ± 23.36	68.55 ± 1.06	1169.37 ± 57.78	43.24 ± 0.40	143.44 ± 6.88	2.29 ± 0.05	8.09 ± 0.27	1.82 ± 0.04	10.57 ± 0.02	0.45 ± 0.03	1.36 ± 0.03	0.48 ± 0.02	1.03 ± 0.01	0.42 ± 0.00	0.83 ± 0.07	< LOQ[b]	0.22 ± 0.01	0.49 ± 0.00	0.38 ± 0.00	1.30 ± 0.02	2251.12 ± 62.34
Flour No.01	8.40 ± 0.49	293.57 ± 5.65	21.13 ± 0.54	619.07 ± 3.50	9.39 ± 0.06	66.45 ± 1.87	0.62 ± 0.00	4.61 ± 0.06	1.38 ± 0.01	14.71 ± 0.07	0.92 ± 0.02	5.35 ± 0.03	0.44 ± 0.03	2.78 ± 0.08	1.27 ± 0.01	9.96 ± 0.21	0.54 ± 0.00	3.13 ± 0.02	0.28 ± 0.01	0.32 ± 0.02	0.96 ± 0.04	1064.94 ± 6.54
Flour No.02	24.12 ± 0.31	405.61 ± 14.66	34.13 ± 1.70	655.41 ± 12.59	17.61 ± 0.17	63.93 ± 0.31	1.60 ± 0.07	8.96 ± 0.13	3.83 ± 0.02	21.32 ± 0.71	2.01 ± 0.08	5.56 ± 0.16	1.10 ± 0.02	3.99 ± 0.02	2.61 ± 0.05	11.13 ± 0.17	1.17 ± 0.02	3.02 ± 0.00	0.20 ± 0.00	0.24 ± 0.02	0.71 ± 0.03	1266.86 ± 18.04
Flour No.03	1.46 ± 0.02	13.31 ± 0.78	1.57 ± 0.01	19.25 ± 0.97	0.92 ± 0.01	1.64 ± 0.08	0.19 ± 0.01	0.84 ± 0.06	0.10 ± 0.00	1.07 ± 0.07	< LOQ[b]	< LOQ[b]	0.18 ± 0.00	0.36 ± 0.01	0.11 ± 0.00	0.34 ± 0.01	< LOQ[b]	< LOQ[b]	< LOQ[b]	< LOQ[b]	< LOQ[b]	41.34 ± 1.32
Flour No.04	20.11 ± 0.35	355.50 ± 7.84	32.44 ± 0.99	671.42 ± 5.33	14.99 ± 0.21	60.73 ± 0.59	0.33 ± 0.04	6.03 ± 0.10	1.83 ± 0.06	14.94 ± 0.05	0.86 ± 0.02	4.01 ± 0.06	0.40 ± 0.02	1.85 ± 0.05	0.96 ± 0.02	4.84 ± 0.23	0.40 ± 0.00	1.67 ± 0.06	0.45 ± 0.03	0.43 ± 0.01	1.24 ± 0.03	1188.11 ± 8.97
Hulled Grain No.01	94.26 ± 2.52	546.21 ± 4.97	94.40 ± 1.33	982.43 ± 2.63	48.16 ± 0.89	109.96 ± 5.36	5.74 ± 0.11	13.62 ± 0.04	7.70 ± 0.05	42.76 ± 1.16	3.36 ± 0.11	11.51 ± 0.27	3.33 ± 0.09	11.37 ± 0.27	7.28 ± 0.11	44.12 ± 0.43	3.06 ± 0.12	11.54 ± 0.23	0.98 ± 0.07	0.79 ± 0.01	2.36 ± 0.07	2041.29 ± 7.96
Hulled Grain No.02	30.9 ± 0.03	31.55 ± 0.60	4.02 ± 0.07	20.32 ± 3.90	3.23 ± 0.08	5.10 ± 0.02	0.30 ± 0.00	1.34 ± 0.06	0.36 ± 0.02	1.96 ± 0.01	0.36 ± 0.01	0.76 ± 0.01	0.12 ± 0.01	0.25 ± 0.01	0.20 ± 0.00	0.62 ± 0.00	< LOQ[b]	0.18 ± 0.02	< LOQ[b]	< LOQ[b]	0.23 ± 0.02	101.84 ± 3.95
Hulled Grain No.03	37.18 ± 0.02	432.60 ± 9.87	70.79 ± 0.38	1002.23 ± 14.94	58.16 ± 2.02	142.59 ± 2.01	0.63 ± 0.05	4.72 ± 0.08	1.53 ± 0.04	9.80 ± 0.05	0.69 ± 0.01	2.05 ± 0.02	0.51 ± 0.01	2.21 ± 0.07	0.79 ± 0.01	4.49 ± 0.23	0.10 ± 0.05	1.00 ± 0.00	0.42 ± 0.01	0.86 ± 0.04	3.71 ± 0.08	1769.86 ± 30.02
Hulled Grain No.04	4.62 ± 0.15	17.12 ± 0.78	5.18 ± 0.11	35.19 ± 0.81	2.66 ± 0.03	1.36 ± 0.02	0.55 ± 0.00	1.22 ± 0.00	0.74 ± 0.00	1.35 ± 0.01	0.27 ± 0.01	0.42 ± 0.00	0.29 ± 0.00	0.26 ± 0.03	0.46 ± 0.02	0.67 ± 0.01	< LOQ[b]	0.17 ± 0.01	< LOQ[b]	< LOQ[b]	< LOQ[b]	72.45 ± 1.81
Pearl Grain No.01	9.60 ± 0.24	63.39 ± 5.72	4.34 ± 0.57	33.70 ± 6.62	3.17 ± 0.07	2.69 ± 0.11	1.53 ± 0.05	6.20± 0.02	1.99 ± 0.03	10.06 ± 0.23	0.69 ± 0.01	1.83 ± 0.01	1.11 ± 0.02	3.75 ± 0.06	1.42 ± 0.04	6.61 ± 0.15	0.32 ± 0.01	1.56 ± 0.08	< LOQ[b]	< LOQ[b]	0.21 ± 0.00	148.47 ± 9.62
Pearl Grain No.02	1.01 ± 0.00	14.06 ± 0.55	1.83 ± 0.01	37.14 ± 1.61	1.92 ± 0.04	6.50 ± 0.04	< LOQ[b]	< LOQ[b]	< LOQ[b]	0.38 ± 0.01	< LOQ[b]	< LOQ[b]	< LOQ[b]	< LOQ[b]	< LOQ[b]	< LOQ[b]	< LOQ[b]	< LOQ[b]	< LOQ[b]	< LOQ[b]	< LOQ[b]	62.84 ± 2.25
Pearl Grain No.03	31.26 ± 1.51	245.57 ± 2.15	39.48 ± 0.24	410.91 ± 10.48	19.81 ± 0.30	43.74 ± 1.66	1.38 ± 0.06	5.02 ± 0.04	2.11 ± 0.01	9.15 ± 0.03	0.28 ± 0.00	1.94 ± 0.03	0.67 ± 0.01	1.69 ± 0.02	0.83 ± 0.02	3.48 ± 0.06	0.03 ± 0.01	0.98 ± 0.03	0.30 ± 0.00	0.43 ± 0.01	1.05 ± 0.03	819.42 ± 16.76
Pearl Grain No.04	10.37 ± 0.38	96.73 ± 7.00	9.89 ± 0.53	149.81 ± 10.16	0.82 ± 0.02	13.16 ± 0.06	0.50 ± 0.05	2.78 ± 0.05	0.75 ± 0.02	3.67 ± 0.12	0.20 ± 0.01	0.77 ± 0.03	0.45 ± 0.01	1.00 ± 0.01	0.29 ± 0.03	0.26 ± 0.02	< LOQ[b]	0.16 ± 0.01	0.16 ± 0.00	0.16 ± 0.01	0.31 ± 0.01	291.94 ± 12.53
Pearl Grain No.05	10.72 ± 0.76	66.26 ± 4.21	10.09 ± 0.33	105.82 ± 4.79	2.26 ± 0.14	11.77 ± 0.16	1.18 ± 0.02	3.10 ± 0.05	1.27 ± 0.01	3.92 ± 0.06	0.43 ± 0.00	0.83 ± 0.01	0.69 ± 0.01	1.03 ± 0.02	0.45 ± 0.01	0.30 ± 0.04	< LOQ[b]	< LOQ[b]	< LOQ[b]	< LOQ[b]	0.27 ± 0.01	218.39 ± 9.22

[a] Standard for quantification, [b] LOQ: Lower of quantification.

*The quantification of barley phenolamides was analyzed in two independent samples.

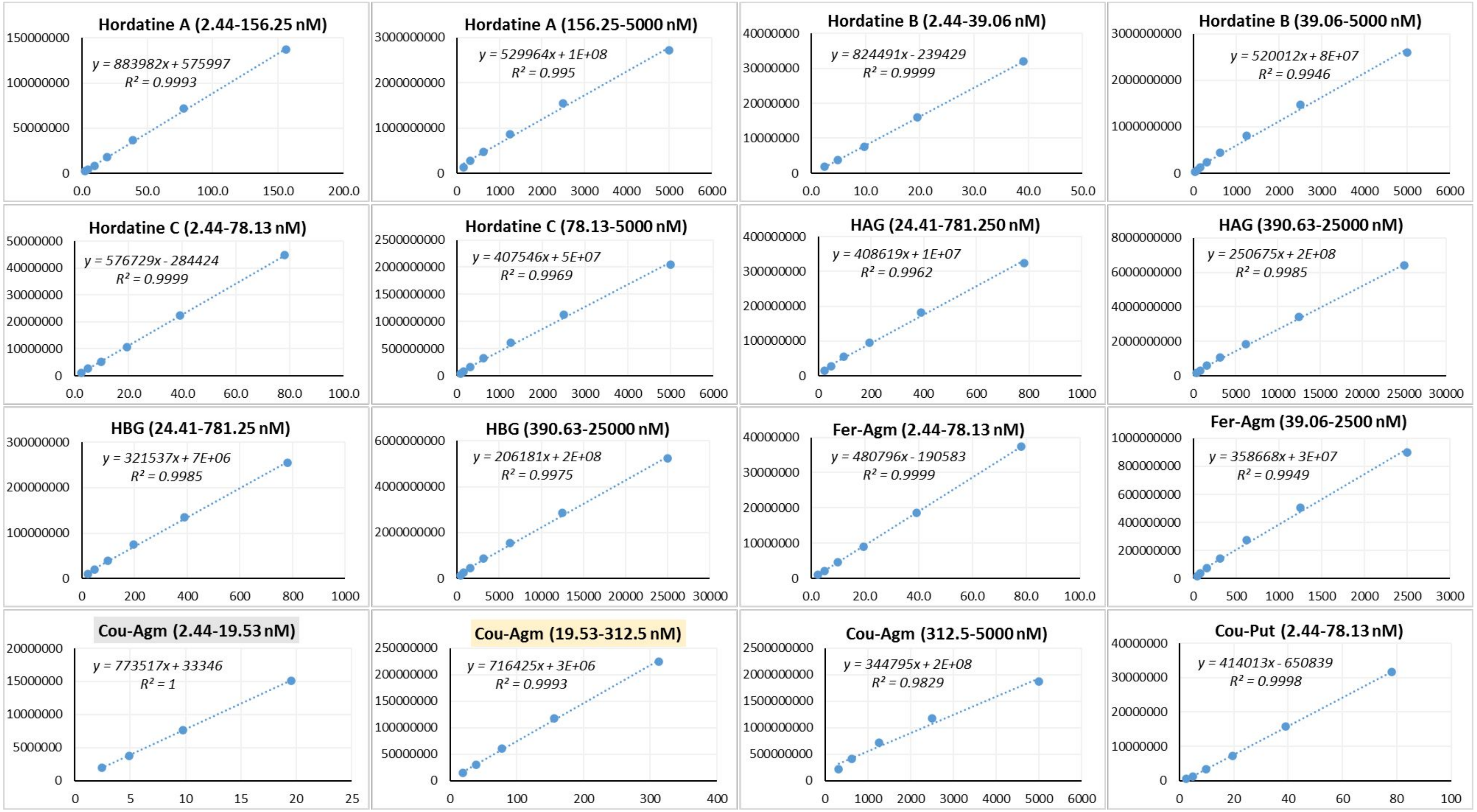

Figure S1. The standard curves of Hordatines A–C, glycosylated hordatines A and B, Cou-Agm, Fer-Agm, and Cou-Put.

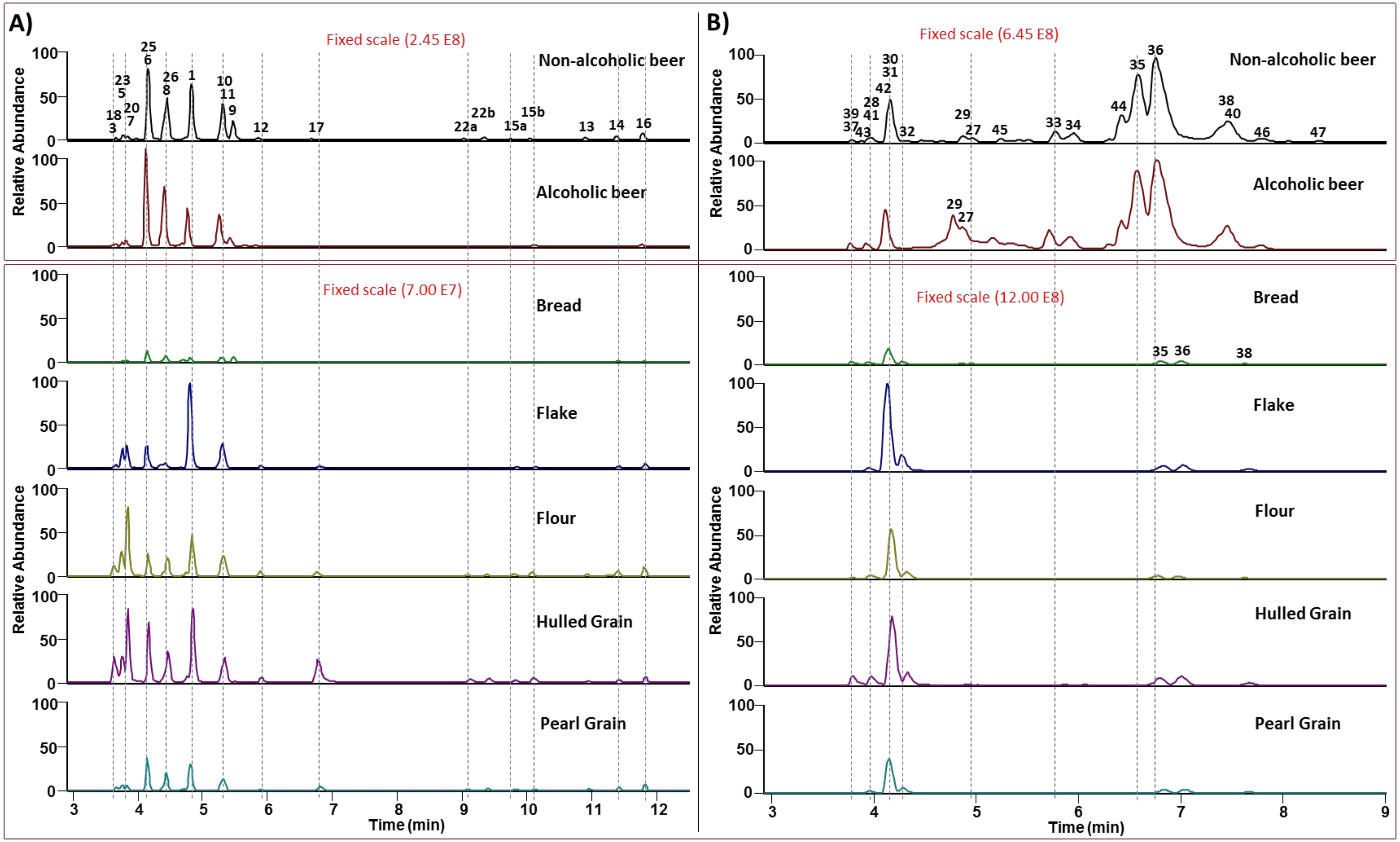

Figure S2. Chemical profile of phenolamides in barley-based products (selected ions with fixed scale); **A**) Agmatine and spermidine conjugates, **B**) Hordatines. The name of compounds corresponding to the numbers are present in the main manuscript (Figures 2, 5 and 9).
